# The MicroRNA and MessengerRNA Profile of the RNA-Induced Silencing Complex in Human Primary Astrocyte and Astrocytoma Cells

**DOI:** 10.1371/journal.pone.0013445

**Published:** 2010-10-18

**Authors:** Joanna J. Moser, Marvin J. Fritzler

**Affiliations:** Department of Biochemistry and Molecular Biology, Faculty of Medicine, University of Calgary, Calgary, Alberta, Canada; City of Hope National Medical Center, United States of America

## Abstract

**Background:**

GW/P bodies are cytoplasmic ribonucleoprotein-rich foci involved in microRNA (miRNA)-mediated messenger RNA (mRNA) silencing and degradation. The mRNA regulatory functions within GW/P bodies are mediated by GW182 and its binding partner hAgo2 that bind miRNA in the RNA-induced silencing complex (RISC). To date there are no published reports of the profile of miRNA and mRNA targeted to the RISC or a comparison of the RISC-specific miRNA/mRNA profile differences in malignant and non-malignant cells.

**Methodology/Principal Findings:**

RISC mRNA and miRNA components were profiled by microarray analysis of malignant human U-87 astrocytoma cells and its non-malignant counterpart, primary human astrocytes. Total cell RNA as well as RNA from immunoprecipitated RISC was analyzed. The novel findings were fourfold: (1) miRNAs were highly enriched in astrocyte RISC compared to U-87 astrocytoma RISC, (2) astrocytoma and primary astrocyte cells each contained unique RISC miRNA profiles as compared to their respective cellular miRNA profiles, (3) miR-195, 10b, 29b, 19b, 34a and 455-3p levels were increased and the miR-181b level was decreased in U-87 astrocytoma RISC as compared to astrocyte RISC, and (4) the RISC contained decreased levels of mRNAs in primary astrocyte and U-87 astrocytoma cells.

**Conclusions/Significance:**

The observation that miR-34a and miR-195 levels were increased in the RISC of U-87 astrocytoma cells suggests an oncogenic role for these miRNAs. Differential regulation of mRNAs by specific miRNAs is evidenced by the observation that three miR34a-targeted mRNAs and two miR-195-targeted mRNAs were downregulated while one miR-195-targeted mRNA was upregulated. Biological pathway analysis of RISC mRNA components suggests that the RISC plays a pivotal role in malignancy and other conditions. This study points to the importance of the RISC and ultimately GW/P body composition and function in miRNA and mRNA deregulation in astrocytoma cells and possibly in other malignancies.

## Introduction

GW bodies (GWB, glycine- and tryptophan-rich cytoplasmic processing bodies; also known as mammalian processing (P) bodies or Dcp containing bodies, hereafter referred to as GW/P bodies) are cytoplasmic foci in mammalian cells enriched in the GW182 protein, which is characterized by multiple glycine (G) and tryptophan (W) repeats and a carboxyl terminal classical RNA binding domain [1]. GW/P bodies have been shown to provide the appropriate microenvironment for the RNA induced silencing complex (RISC) and critical steps in the RNA interference (RNAi) pathway [2], [3]. Key components of GW/P bodies include Dicer, human Argonaute 2 (hAgo2), and microRNAs (miRNA) (reviewed in [4]–[7]). In addition, GW182 co-localizes with 5′→3′ mRNA decay factors, CCR4, Dcp1, LSm4 and XRN1 [8]–[11] implicating GW/P bodies as sites for messenger RNA (mRNA) processing and degradation [5], [12]. It is currently thought that silencing and degrading factors are partitioned to GW/P bodies to increase the efficiency of post-transcriptional regulation and to prevent the inadvertent degradation of functional mRNA [13].

RNAi is the key pathway involved in the post-transcriptional silencing of >50% of all mRNAs in cells and tissues from a variety of organisms [14]. RNAi is mediated by endogenous double-stranded RNA (dsRNA) precursors termed pre-miRNA that are rapidly processed into miRNA duplexes of 18–22 nucleotides in length by Dicer, a dsRNA-specific endonuclease [3]. These small RNA duplexes are then incorporated into the RISC where the passenger miRNA strand is dissociated by cleavage, degradation or a bypass mechanism [15]. The remaining guide miRNA strand subsequently activates the RISC by interacting with hAgo2 [16], [17]. The RISC then recruits one or more heteromeric protein complexes (e.g. GW182 and RCK/p54) to associate with the mRNA leading to the formation of GW/P bodies. Depending on the degree of complementarity between the guide-strand miRNA and its target mRNA, the augmented RISC then initiates post-transcriptional inhibition of gene expression through translational repression [4], [18]. Of considerable importance, each miRNA is predicted to regulate hundreds of different target mRNAs while a single mRNA has the potential to be regulated by dozens of different miRNAs. GW/P bodies are formed in response to RNA-mediated gene silencing [19], [20] and are regarded as sites for miRNA-mediated mRNA silencing [4], [9], although some studies have shown that the process of active RNAi can occur in the absence of conventional microscopically visible GW/P bodies [9], [21]. Further, GW/P body protein components and binding partners, GW182 and hAgo2, are co-factors in miRNA-mediated translational repression and mRNA degradation [22] whereby the C-terminal domain of GW182 is essential for miRNA function [23], the RRM domain of GW182 has been shown to contribute to miRNA-mediated silencing of mRNA [24] and the C-terminal domain of hAgo2 must bind to the GW-rich regions of GW182 to mediate silencing [25].

To date, miRNA have been shown to have an effect on the development of many cancers [26]–[29] and it is now understood that greater than half of the annotated human miRNA genes are located in chromosomal regions frequently displaying amplification, deletion, or translocation in human cancers [30]. In cycling/proliferating cells (S/G_2_), such as cancer cells, miRNAs are generally thought to repress mRNA translation [31]. In addition, microRNAs are aberrantly expressed in a wide variety of human cancers, including but not limited to those originating in ovarian, breast, lung, gastrointestinal and brain tissues where they are thought to play important roles by regulating the expression of various oncogenes and tumor suppressors [28], [32]–[37]. When miRNAs are downregulated, they themselves can act as tumor suppressors [38] or, when overexpressed, act as oncogenes (referred to as oncomirs) [26], [39], [40] possibly through dysregulation of the cell cycle leading to the formation of tumors.

Of more relevance to the current study, emerging evidence has demonstrated that distinct patterns of microRNA expression were found in glioblastomas and indicated that microRNAs play key roles in maintaining malignant characteristics of stem cell behavior, cell proliferation and invasion, and angiogenesis (reviewed in [41]). For example, recent studies have shown that miRNA-451 regulated signaling is a key in stress adaptation of glioma cells [42] and dicer-regulated miRNAs-222 and -339 promote resistance to cytotoxic T-lymphocytes by down-regulating ICAM-1 [43]. Other evidence has demonstrated that p27(Kip1) is a direct target for miRNAs 221 and 222, and may promote aggressive growth characteristics of glioblastomas [44], and miR-26a-mediated PTEN repression in a murine glioma model enhanced *de novo* tumor formation and indicates a new epigenetic mechanism for PTEN regulation in gliomas [45].

In this study, we used miRNA microarray and gene expression microarray analyses to identify for the first time the miRNA and mRNA components of the RISC in U-87 astrocytoma cells and primary human astrocytes as compared to the global cellular miRNA and mRNA in these cells. We found that miRNAs are highly enriched in primary astrocyte RISC compared to U-87 astrocytoma RISC and that both cell lines contain unique RISC miRNA profiles compared to their respective global cellular miRNA. Specifically, U-87 astrocytoma RISC and primary astrocyte RISC share some common miRNAs but also contain their own unique subset of miRNAs. Further, RISC were found to contain mostly downregulated mRNAs in both primary astrocyte and U-87 astrocytoma cells. Taken together, our data suggest that RISC miRNA and mRNA components are different from the miRNA/mRNA profile of the global cellular milieu and key differences between malignant and non-malignant cells may point to novel diagnostic and therapeutic interventions.

## Materials and Methods

### Cells

Primary human cerebral cortex astrocyte cells (used between passages 4–6, cat# 1800, ScienCell Research Laboratories, Carlsbad, CA) were cultured under 5% CO_2_ in astrocyte medium containing 2% fetal bovine serum, 1% astrocyte growth supplement and 1% penicillin/streptomycin (ScienCell Research Laboratories). Adherent human U-87 MG astrocytoma/glioblastoma cells (used between passages 18–22, cat# HTB-14, American Type Culture Collection (ATCC), Rockville, MD) were cultured under 5% CO_2_ in DMEM F12+1% L-glutamine (2 mM) (Cambrex, Walkersville, MD) supplemented with 10% fetal bovine serum (Gibco, Burlington, ON, Canada), 1% penicillin-streptomycin (Gibco), 1% sodium pyruvate (1 mM) (Gibco) and 1% non-essential amino acids (0.1 mM) (Gibco) at 37°C.

### Ribonucleoprotein (RNP) Lysate Preparation

Primary astrocytes and U-87 astrocytoma cells were cultured as described above in multiple 150 cm^2^ tissue culture flasks, harvested, washed in sterile RNase-free ice-cold phosphate buffered saline (PBS: Ambion, Streetsville, ON, Canada), pooled and pelleted by centrifugation at 196 *g* for 5 min at 4°C as previously described in detail [46]. PBS was aspirated from the cell pellets and 1 equal volume of freshly prepared RNase-free ice-cold polysome lysis buffer (10 mM HEPES pH 7.0, 100 mM KCl, 5 mM MgCl_2_, 25 mM EDTA pH 8.0, 0.5% IGEPAL, complete EDTA-free protease inhibitors, 2 mM DTT, 50 U/ml RNase OUT, 50 U/ml Superase IN) was added to the cell pellets [47]. Whole cell RNP lysates were vortexed, set on ice for 3 minutes and then transferred to −80°C until use. To isolate the cellular RNP proteins, the lysates were thawed on ice, sheared by passing them through a syringe fitted with a 21G 1.5-inch needle and centrifuged in a tabletop microfuge at 16,000 *g* for 10 min at 4°C [11], [46]. The supernatant was removed, transferred to a new centrifuge tube and re-centrifuged at 16,000 *g* for 10 min at 4°C to facilitate removal of the lipid layer overlying the supernatant. The protein concentration in the cellular RNP lysate was quantitated using a NanoVue spectrophotometer (GE Healthcare Life Sciences, Baie d'Urfe, QC, Canada) and stored at −80°C. The protein concentration of the cytoplasmic RNP lysates was 12 mg/ml for primary astrocytes and 11 mg/ml for U-87 astrocytoma.

### RISC RNA Immunoprecipitation (RISC-RIP)

Human serum 18033 (Mitogen Advanced Diagnostics Laboratory, Calgary, AB), was used as the source of antibodies that were covalently coupled to protein A-Sepharose beads as previously described [11], [46], [48]. We have previously shown that human serum 18033 immunoprecipitated both GW182 and hAgo2 proteins [46]. Prior to immunoprecipitation (IP), 150 µl of the antibody-coupled protein A-Sepharose beads for each IP reaction was washed five times with NT2 buffer (50 mM Tris-HCl pH 7.4, 150 mM NaCl, 1 mM MgCl_2_, 0.05% IGEPAL) at room temperature. Washed antibody-coupled beads were resuspended in 1000 µl of freshly prepared ice-cold NET2F buffer (850 µl NT2 buffer, 10 µl 0.1 M DTT, 30 µl 0.5 M EDTA pH 8.0, 50 µl of 50 mg/ml ultrapure bovine serum albumin, 5 µl RNase OUT, 5 µl Superase IN) and mixed by inversion. Immunoprecipitation of the RISC-RNP complexes was accomplished by adding 200 µl of the cellular RNP lysates to the antibody-coupled beads in NET2F buffer. IP reactions were incubated overnight at 4°C on a rotator. The beads were then centrifuged at 82 *g* for 30 seconds at 4°C, the supernatant removed and the RISC-RNP complex-antibody-beads were washed six times in 1 ml of ice-cold NT2 buffer. RISC-RNP complexes were dissociated from the antibody-beads by SDS-TE heat denaturation by resuspension of washed beads in 100 µl NET2F buffer and 100 µl of SDS-TE buffer (20 mM Tris-HCl pH 7.5, 2 mM EDTA pH 8.0, 2% SDS) and incubation at 55°C for 30 min [47]. Total RNA enriched in small RNAs was isolated from the RISC-RNP complex using the mirVana kit (Ambion) according to the procedures outlined in the manufacturer's protocol. RNA samples were stored at −80°C until required for analysis. The total RNA concentration of the RISC-RNP complex was 0.108 µg/µl for primary astrocytes and 0.318 µg/µl for U-87 astrocytoma.

### µParaflo MicroRNA Microarray Assay

Microarray assays of global miRNA and RISC-immunoprecipitated miRNA in primary human astrocytes and U-87 astrocytoma cells was outsourced to LC Sciences (Houston, TX). To isolate global miRNA, total RNA was isolated from primary human astrocytes (ScienCell) and U-87 astrocytoma cells (ATCC) using the mirVana kit (Ambion). The global total RNA concentration was 4.10 µg/µl for primary astrocytes and 2.49 µg/µl for U-87 astrocytoma cells. RISC-specific total RNA was isolated as described above and RNA integrity was assessed with an Agilent Bioanalyzer 2100 (Foster City, CA). The microarray assay was performed using samples containing 5 µg total RNA, which were fractionated using a YM-100 Microcon centrifugal filter (Millipore, Billerica, MA) to isolate small RNAs (<300 nt). Small RNAs were 3′-extended with a poly(A) tail using poly(A) polymerase. An oligonucleotide tag was then ligated to the poly(A) tail for later fluorescent dye staining with Cy5.

Hybridizations were performed overnight on a µParaflo microfluidic chip using a micro-circulation pump (Atactic Technologies, Houston, TX) [49]. Each detection probe on the microfluidic chip consisted of a chemically modified nucleotide coding segment complementary to 6211 target human miRNAs (Sanger miRBase version 11, http://microrna.sanger.ac.uk/sequences/) or control RNAs (array hybridization controls and single-base mismatch targets), and a polyethylene glycol spacer segment to extend the coding segment away from the substrate. The detection probes were made by *in situ* synthesis using photogenerated reagent chemistry and were replicated seven times within each microarray chip [50]. The hybridization melting temperatures of the probes were balanced by chemical modifications of the detection probes. Hybridization was performed at 34°C using 100 µL of 6xSSPE buffer (0.90 M NaCl, 60 mM Na_2_HPO_4_, 6 mM EDTA pH 6.8) containing 25% formamide. After washing, the hybridized fluorescent Cy5 signals were detected with a laser scanner (GenePix 4000B, Molecular Devices, Sunnyvale, CA) and digitized by Array-Pro image analysis software (Media Cybernetics, Bethesda, MD).

Data were further processed by subtracting the background followed by signal normalization with a LOWESS (locally weighted regression) filter [51]. For a miRNA transcript to be classified as reliably detectable, it had to satisfy the following criteria: overall signal intensity >3× background standard deviation, spot coefficient of variation <0.5, and >50% of the repeated probes had signal intensities above detection level. The data were log_2_ transformed and the p-values of the t-test were calculated. Differentially detected miRNA signals with a p value of less than 0.01 were considered significant (p<0.01). Heat maps were generated for the differentially expressed miRNAs with p<0.01.

### Gene expression microarray

Gene expression microarray analyses of global mRNA and RISC-immunoprecipitated mRNA in primary human astrocytes and U-87 astrocytoma cells were outsourced to LC Sciences who are partnered with an Affymetrix® Authorized Service Provider, SeqWright DNA Technology Services (Houston, TX). Global and RISC-RNP enriched total RNA were isolated as described above and RNA integrity was assessed by an Agilent Bioanalyzer 2100 (Foster City, CA). Affymetrix's GeneChip IVT Express kit was used for cDNA synthesis and in vitro transcription. The gene expression microarray assay was performed using a 5 µg total RNA sample on an Affymetrix Human Genome U133A 2.0 Array which analyzes the expression level of 18,400 transcripts and variants, including 14,500 well-characterized human genes (Affymetrix, Santa Clara, CA). All procedures and analyses were performed according to the protocol outlined in detail in the GeneChip® Expression Analysis Technical Manual (Affymetrix, http://www.affymetrix.com/support/downloads/manuals/expression_analysis_technical_manual.pdf). Multi-chip expression intensity normalization was performed for all 4 samples on gene expression chips using the robust multichip average (RMA) algorithm [52], which consisted of three steps: background correction, quantile normalization (each performed at the individual probe level), and robust linear model fit using log-transformed intensities (at the probe set level). RMA normalization was used because multiple chips were compared and also because RMA methods allow for better detection of low-abundance genes, which include many genes of interest, such as transcription factors, and signalling proteins. The overall selection criteria were based on expression values larger than a threshold 3.5 in more than 50% of samples.

### Data Availability

The miRNA and mRNA microarray data generated by this study are available in the NCBI Gene Expression Omnibus (GEO) as series accession identifier GSE21514.

### Functional Network Analysis

Functional profiling was performed by employing the computational gene network prediction tool Ingenuity Pathway Analysis version 8.5 (IPA, Ingenuity® Systems; http://www.ingenuity.com) on all significantly expressed (p<0.01) miRNAs with log2 fold change greater than or equal to 2 and mRNAs with a log2 fold change greater than or equal to 2.5. For biological pathway connections in U-87 astrocytoma RISC, miRNAs with a log2 fold change greater than or equal to 3 and mRNAs with a log2 fold change greater than or equal to 2 were used. IPA mapped the uploaded microarray datasets into a global molecular network developed from a literature-supported Ingenuity Pathways Knowledge Base, and then generated networks that represent the molecular relationships between miRNA and mRNA. Canonical pathways were selected and overlayed onto the biological pathway based on known biological significance from the most highly overlapping pathways. MRNAs and miRNAs without connections to other molecules or pathways were removed from the final figure. The biological functions significantly associated with the genes in the networks are provided by the Ingenuity Pathways Knowledge Base and scored employing Fischer's exact test with a p<0.01.

### cDNA Synthesis and Quantitative Reverse Transcription Polymerase Chain Reaction (qRT-PCR)

Global and RISC-RNP-enriched total RNA was extracted and quantitated as described above. Poly-A tailing of the miRNA in the total RNA sample and cDNA synthesis was performed using the NCode VILO miRNA cDNA synthesis kit (Invitrogen, Burlington, ON, Canada) according to the manufacturer's instructions. In brief, 1 µg of total RNA was added to 4 µl of 5X Reaction Mix and 2 µl of 10X SuperScript Enzyme mix up to a total volume of 20 µl. Reactions were mixed by pipeting and incubated at 37°C for60 min. The reactions were terminated at 95°C for 5 min and stored at −20°C until use in the qRT-PCR reaction. The cDNA was diluted with nuclease free water to give a DNA concentration of ∼375 ng/µl and qRT-PCR reactions performed using the EXPRESS SYBR GreenER Universal qRT-PCR kit (Invitrogen). Each 20 µl qRT-PCR reaction contained 10 µl of EXPRESS SYBR GreenER qPCR SuperMix Universal, 0.4 µl of 10 µM miRNA-specific forward primer or 10 µM mRNA-specific forward primer (listed in Table S1), 0.4 µl of 10 µM Universal reverse qPCR primer or 10 µM mRNA-specific reverse primer (listed in Table S1), 1 µl of 1∶10,000 diluted fluorescein calibration dye and 1 µl of diluted cDNA up to a final volume of 20 µl with nuclease-free water. The qRT-PCR reactions were performed in triplicate with a BioRad MyiQ Single Color Real-Time PCR detection system iCycler programmed as follows: 1 cycle at 50°C for 2 min for UDG incubation; 1 cycle at 95°C for 2 min; 40 cycles of 95°C for 15 seconds and 60°C for 1 min, followed by a melting curve analysis at 95°C for 1 min and 60°C for 1 min and 10 sec. The cycle threshold (Ct) values, corresponding to the PCR cycle number at which fluorescence emission reaches a threshold above baseline emission, were determined and the mRNA and miRNA levels calculated using the comparative 2^-ΔΔCt^ method. A comparison of the relative mean fold increase in gene expression between two housekeeping genes, beta-actin and 80S RNA was examined to determine the most stable housekeeping gene. Due to smaller standard deviations between the 2^-ΔΔCt^ over 3 independent experiments, the beta-actin gene was used as the reference marker for gene expression normalization. For analysis of miRNA by qRT-PCR, RNU44 and RNU6B controls were examined to determine the most stable small RNA endogenous control. Due to smaller standard deviations between the 2^-ΔΔCt^ values over 3 independent experiments, RNU6B was used as the reference marker for miRNA expression normalization.

## Results and Discussion

### miRNAs are highly enriched in primary astrocyte RISC compared to U-87 astrocytoma RISC

**Figure 1:**
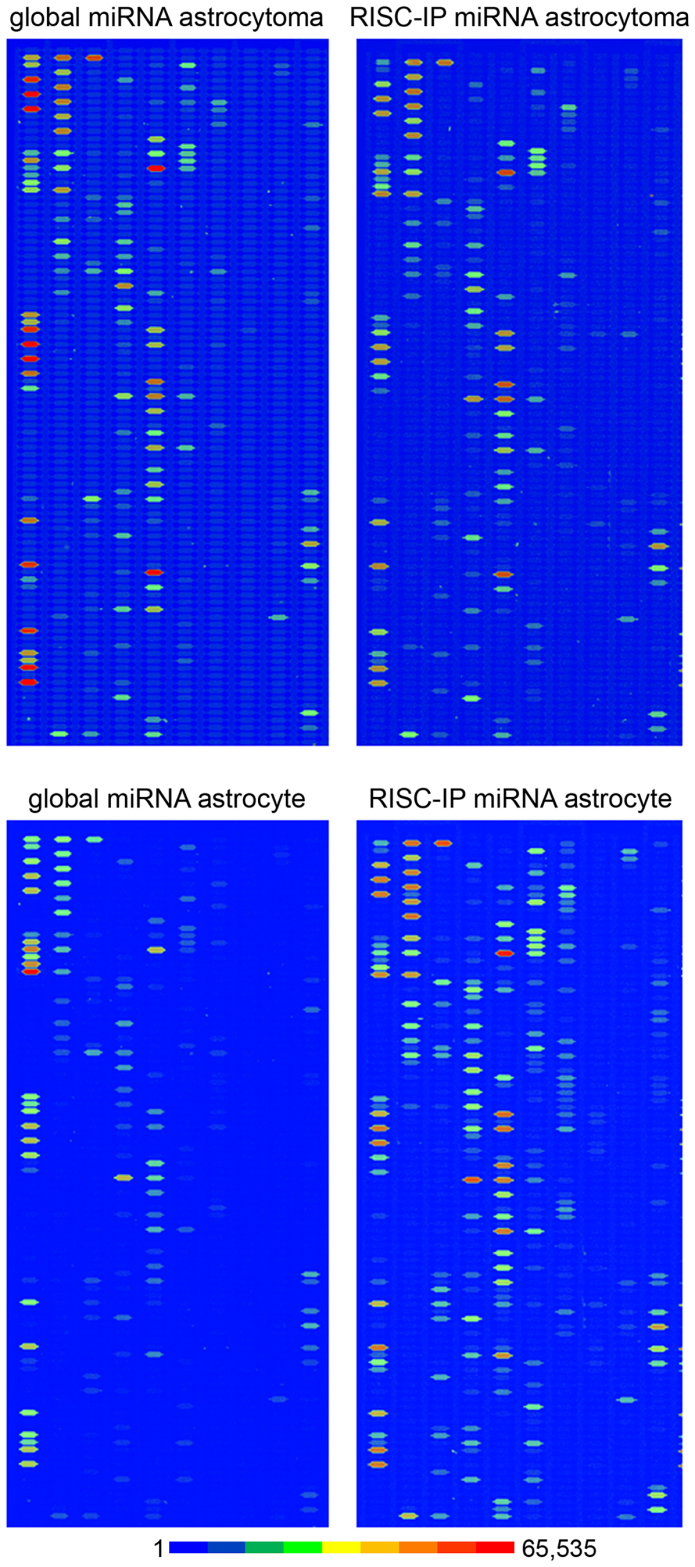
MiRNA microarray images of global and RISC-immunoprecipitated (RISC-IP) miRNA from human U-87 astrocytoma and primary human astrocyte cells. The representative chip images are displayed in pseudo colors to expand the visual dynamic range. As signal intensity increases from 1 to 65,535, the corresponding color changes from blue to green, to yellow and to red.

MiRNA microarray analysis was undertaken to examine the spectrum of cellular miRNAs in primary human astrocyte and human U-87 astrocytoma cells and then compared to those associated with the RISC of the respective cells. Several key observations were made from the examination of the four miRNA microarray data domains. First, comparison of U-87 astrocytoma cell and primary astrocyte global miRNA at equivalent RNA concentrations showed that the signal intensity of global miRNAs in the U-87 astrocytoma cells were generally increased compared to primary astrocytes (Figure 1, top and bottom left) suggesting that astrocytoma cells may have an increased number of individual miRNAs compared to the number in normal astrocytes. In addition, the global miRNA profile of astrocytoma cells was enriched in, and contained a greater variety of, miRNA than primary astrocytes. Second, comparison of global miRNA and RISC-immunoprecipitated (RISC-IP) miRNA in astrocytoma cells demonstrated that the signal intensity of the same miRNA species did not vary between the RISC-enriched population and that of the global miRNA population (Figure 1, top left and right) suggesting that astrocytoma cell RISC are not as highly enriched in specific miRNAs and display the same relative signal intensity and contain many of the same miRNAs as astrocytoma cells. Third, comparison of global miRNA and RISC-IP miRNA revealed that the signal intensity and number of miRNAs of the RISC-IP miRNA was remarkably increased in primary astrocytes compared to that of the global astrocyte miRNAs (Figure 1, bottom left and right), suggesting that a greater number of miRNAs are enriched in primary astrocyte RISCs. Fourth, comparison of astrocytoma and astrocyte RISC-IP miRNA indicated that the number of miRNA species and the signal intensity of miRNAs present in the primary astrocyte RISC-IP were increased (Figure 1, right top and bottom) indicating that primary astrocyte RISC may be enriched in a relatively more diverse population of miRNAs and contain a greater number of individual miRNAs than U-87 astrocytoma RISC. These results suggested that depletion or deregulation of the specific miRNA population in RISC could potentially play an important role in the maintenance of the malignant state.

The signal probe intensity plot comparing the RISC-IP miRNAs to global miRNAs for U-87 astrocytoma cells and primary astrocytes showed many miRNAs that were equally expressed in cell fractions from both cell types (Figure S1). However, in both cell types, there was a subset of miRNAs that had higher signal intensity in RISC (Figure S1) and lower signal intensity in RISC (Figure S1). Furthermore, both cell types had their own unique miRNA signal distribution pattern suggesting that each cell type had a unique RISC miRNA profile.

### RISC miRNA profile in primary human astrocytes

**Figure 2:**
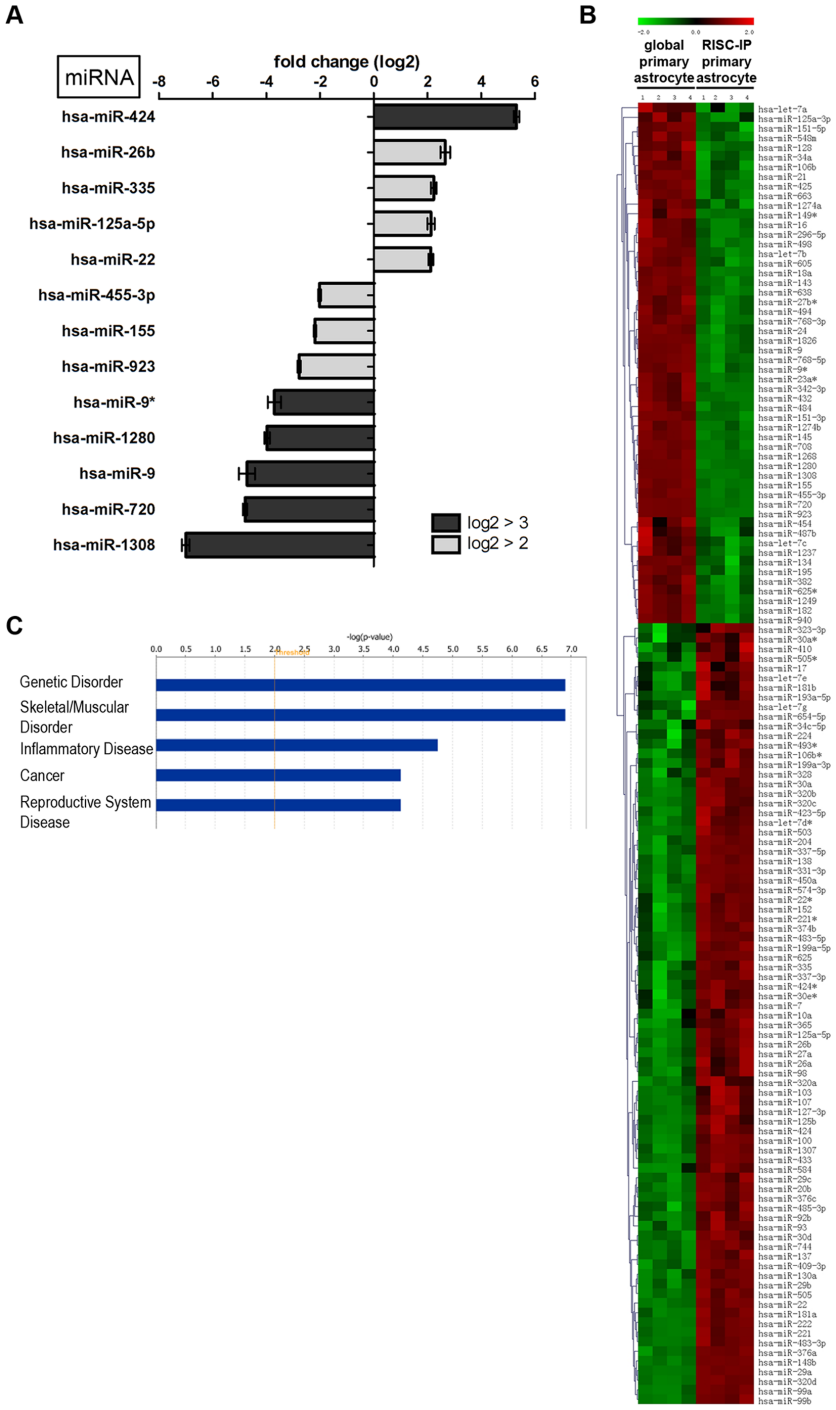
RISC-IP miRNA expression compared to global miRNA expression in primary human astrocytes. (A) MiRNAs with a log2 fold change >2 (p<0.01). MiRNAs with a positive fold change were increased in the RISC compared to the global cellular milieu in primary astrocytes and miRNAs with a negative fold change are decreased in RISC compared to the global cellular milieu in primary astrocytes. (B) Hierarchical cluster heatmap showing all significantly expressed miRNAs (p<0.01) in four independent samples/cell types. Each row shows the relative expression level for a single miRNA and each column shows the expression level for a single sample. The red or green color indicates relative high or low expression, respectively. (C) Disease/disorders associated with the miRNAs differentially expressed in RISC compared to the global cellular milieu of primary astrocytes with a Fisher's exact test p-value threshold set at 0.01 (yellow threshold line) using Ingenuity Pathway Analysis software.

Primary astrocyte RISC contained a unique subset of significantly expressed (p<0.01) miRNAs when normalized to the expression levels of global astrocyte miRNAs (Figure 2). In this study, all significantly expressed miRNAs with a log2 fold change >±2 and a p<0.01 were regarded as miRNAs with significantly different expression. In astrocyte RISC, 13 miRNAs had a log2 fold change greater or equal to ±2 (Figure 2A) and of these, miR-424, miR-22, 125a-5p, 335 and 26b were increased by >2 fold change compared to global miRNA levels (Figure 2A). Notably, miR-424 was increased in RISC by ∼5 fold. MiR-9*, 1280, 9, 720 and 1308 were all decreased in RISC with a log2 fold change below −3 and miR-455-3p, 155 and 923 were decreased between −2 and −3 fold (Figure 2A). Only 13 miRNAs had a fold change >±2, yet many RISC-IP miRNA expression levels could still considered to be significantly different from global miRNA expression levels (p<0.01) (Figure 2B). Significant miRNA expression levels for 4 independent RISC-IP and global samples from primary astrocytes were plotted on a hierarchical clustered heatmap, illustrating that 60% of miRNAs (81 of 135) were increased while the remaining 40% (51/135) were decreased in RISC in primary astrocytes compared to the global cellular miRNA pool (Figure 2B).

Ingenuity Pathway Analysis (IPA) of the 13 key miRNAs with a log2 fold change >±2 in astrocyte RISC predicted that these miRNAs were predominantly associated with genetic disorders and musculoskeletal disorders, however, these specific miRNAs have also been predicted to associate with inflammatory disease, cancer and reproductive system disease (Figure 2C, threshold set at p<0.01 using Fisher's exact test).

### RISC miRNA profile in human U-87 astrocytoma cells

**Figure 3:**
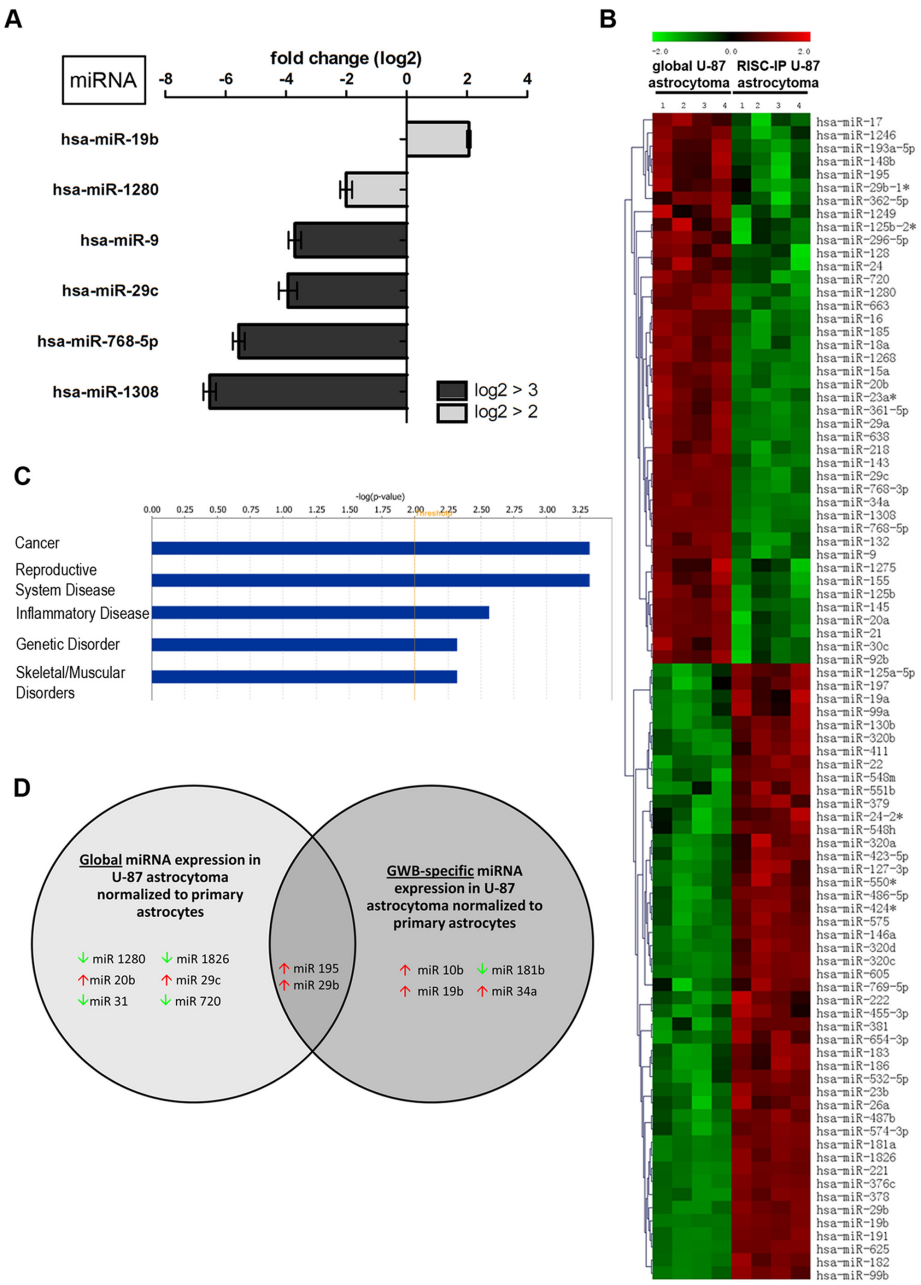
RISC-IP miRNA expression compared to global miRNA expression in human U-87 astrocytoma cells. (A) MiRNAs with a log2 fold change >2 (p<0.01). MiRNAs with a positive fold change are increased in RISC compared to the global cellular milieu in astrocytoma cells and miRNAs with a negative fold change are decreased in RISC compared to the global cellular milieu in astrocytoma cells. (B) Hierarchical cluster heatmap showing all significantly expressed miRNAs (p<0.01) in four independent samples/cell type. Each row shows the relative expression level for a single miRNA and each column shows the expression level for a single sample. The red or green color indicates relative high or low expression, respectively. (C) Disease/disorders associated with the miRNAs differentially expressed in RISC compared to the global cellular milieu of U-87 astrocytoma cells with a Fisher's exact test p-value threshold set at 0.01 (yellow threshold line) using IPA software. (D) MiRNAs in RISC compared to the global cellular fraction in U-87 astrocytoma cells. U-87 astrocytoma cell miRNA expression was normalized to primary astrocytes miRNA expression. MiRNAs displayed have a log2 fold change >3. Green and red arrows indicate decreased and increased miRNA levels respectively.

U-87 astrocytoma cell RISC also contained a unique subset of miRNAs when normalized to the global U-87 astrocytoma cell miRNA levels (Figure 3). As above, miRNAs having levels log2 fold >±2 and a p<0.01 were reported as miRNAs with key differences. In U-87 astrocytoma RISC, only 6 miRNAs had a log2 fold change >±2 (Figure 3A). Of these miRNAs, only miR-19b was increased by >±2 fold compared to global miRNA levels (Figure 3A). MiR-9, 29c, 768-5p and 1308 were all decreased in RISC with a log2 fold change below −3 and miR-1280 was decreased between −2 and −3 fold (Figure 3A). Of interest, in both U-87 astrocytoma and primary astrocyte RISC, miR-9 and miR-1308 were both decreased with the same relative log2 fold change value (Figure 2 and 3). Only 6 miRNAs had a fold change >±2, yet many U-87 astrocytoma RISC-IP miRNA levels were still considered to be significantly different from global miRNA levels (p<0.01) (Figure 3B). Significant miRNA levels for 4 independent RISC-IP and global samples from U-87 astrocytoma cells were plotted on a hierarchical clustered heat map and show that 52.8% of miRNAs (47 of 89) were increased in U-87 astrocytoma cells RISC as compared to the global cellular miRNA pool (Figure 3B). The remaining 47.2% of miRNAs (42 of 89) were decreased in U-87 astrocytoma RISC (Figure 3B). Of note, U-87 astrocytoma RISC contained 46 fewer miRNA species (p<0.01) than primary astrocyte RISC (Figure 2B compared to Figure 3B). In addition, comparison of the population of miRNAs that were increased, U-87 astrocytoma RISC contained 12.8% fewer miRNAs as compared to primary astrocyte RISC (compare Figure 2B and Figure 3B).

IPA of the 6 key miRNAs with a log2 fold change >±2 in U-87 astrocytoma RISC predicted that these miRNAs may be predominantly associated with cancer, however, these specific miRNAs have also been predicted to be associated with inflammatory diseases and genetic disorders (Figure 3C, threshold set at p<0.01 using Fisher's exact test). Interestingly, U-87 astrocytoma RISC miRNAs were predicted to be predominately associated with cancer (Figure 3C), whereas in primary astrocytes, RISC miRNAs were less associated with cancer and more with genetic disorders (Figure 2C). The –log (p-value) scale differs between Figures 2C and 3C due to the number of miRNAs used for IPA analysis (i.e. 13 vs. 6), but functional trend analysis shows that miRNAs associated with inflammatory disease appear to be approximately the same for both U-87 astrocytoma cells and primary astrocytes (compare Figure 2C and Figure 3C).

Upon normalization of significant (p<0.01) U-87 miRNA microarray values to primary astrocyte miRNA microarray values, RISC-specific miRNAs could be identified and separated from miRNAs that were unique to the global cellular milieu (i.e. were not a part of RISC) in U-87 astrocytoma cells (Figure 3D). Unique to RISC in U-87 astrocytoma cells were miR10b, 19b, 34a and 181b. Of this U-87 astrocytoma RISC subset, all were increased with the exception of miR-181b which was decreased when compared to astrocytes (Figure 3D). MiRNAs not present in U-87 astrocytoma RISC included miR-1280, 1826, 31, 720, 20b and 29c all of which were decreased with the exception of miR-20b and 29c which were increased when compared to astrocytes (Figure 3D). MiR-195 and 29b were found in both U-87 astrocytoma RISC and in the cellular milieu and were both increased but with different levels (Figure 3D). Global miR-195 was increased with a log2 fold of 4.491, a difference of −0.977 from RISC expression. On the other hand, global miR-29b was increased by a log2 fold of 6.214 a difference of +1.580 from RISC expression, suggesting that miR-29b has a more significant regulatory role in the global cellular fraction than in the RISC.

**Figure 4:**
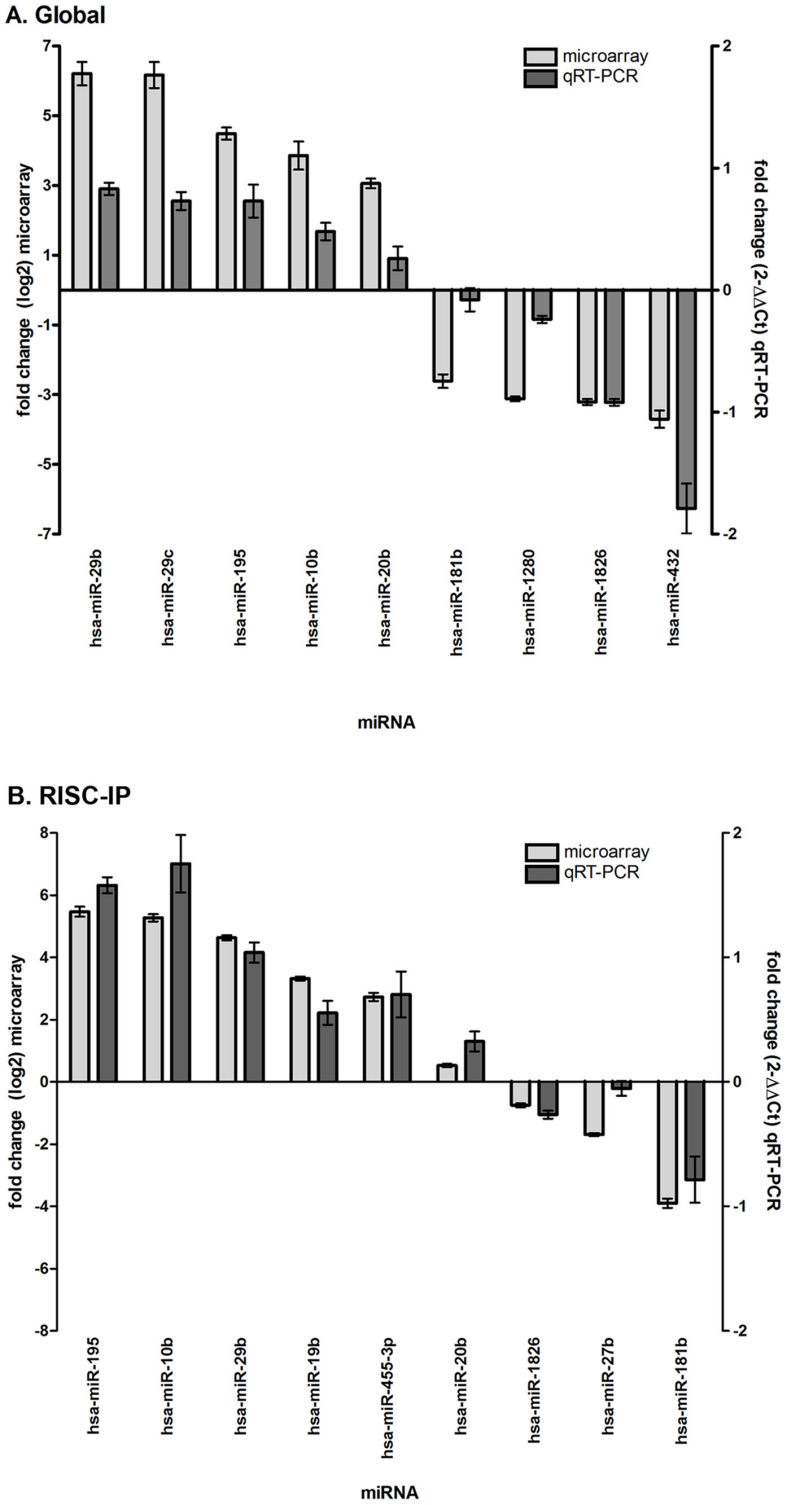
MiRNA microarray validation with qRT-PCR analysis for (A) global miRNA and (B) RISC-specific miRNA. Nine random miRNAs were selected from the miRNA microarray datasets and examined by qRT-PCR. Fold change from the miRNA microarray are given by log2 values (left y-axis, light grey bars). Fold change from the qRT-PCR was determined using the 2^-ΔΔCt^ method and all miRNA expression values were normalized to the RNU6B endogenous control (right y-axis, dark grey bars). Error bars represent the standard deviation of the mean (SD). Note: only the general trend of up-regulation and down-regulation can be compared but the fold change (y-axis) cannot be directly compared between assays due to differences in calculation methods.

The global and RISC-IP miRNA microarray results were subsequently validated by qRT-PCR with miRNA normalized to the endogenous RNU6B control (Figure 4). A total of 18 miRNAs were examined by qRT-PCR for global and RISC-IP miRNA in U-87 astrocytoma cells as normalized to primary astrocytes (Figure 4). The values of fold change cannot be directly compared between microarray and qRT-PCR assays due to differences in calculation methods (log2 for microarray and 2^-ΔΔCt^ for qRT-PCR), yet comparisons could be made based on the general trend of increased or decreased levels (Figure 4). For global miRNA, miR-29b, 29c, 195, 10b and 20b were increased in qRT-PCR analyses and exhibited the same general trend as determined by microarray analysis (Figure 4A). The trend was not as evident for the decreased global miRNAs where there was slightly greater variation in miR-181b, 1280 and 432 qRT-PCR and microarray expression values; however miR-1826 levels coincided between assays (Figure 4A). For RISC-IP miRNA, the trend is similar for both microarray and qRT-PCR assays (Figure 4B).

### Specific miRNA levels are different in U-87 astrocytoma RISC compared to primary astrocyte RISC

**Figure 5:**
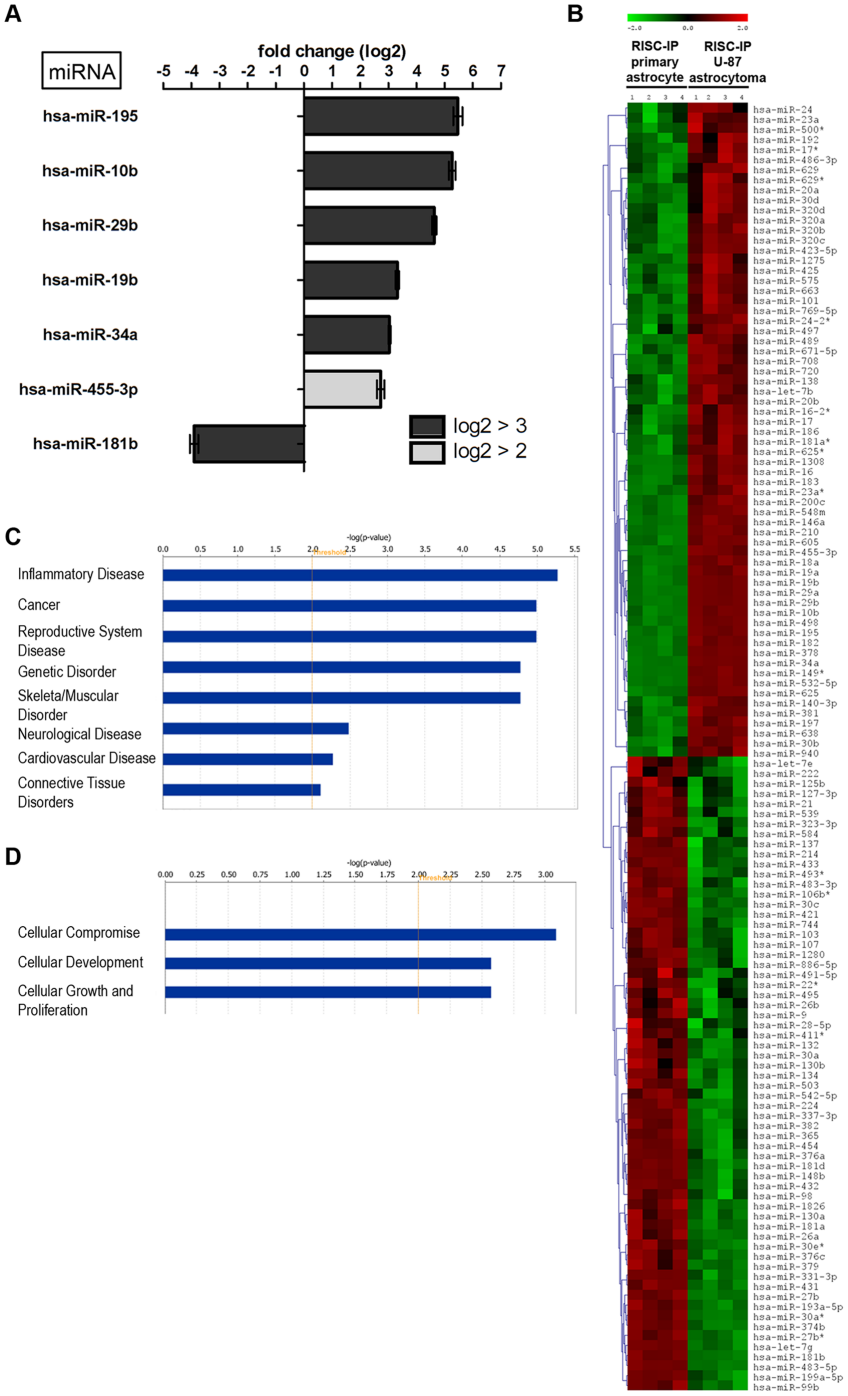
RISC-IP miRNA expression in human U-87 astrocytoma cells compared to primary human astrocytes. (A) MiRNAs with a log2 fold change >2 (p<0.01). MiRNAs with a positive fold change are increased in RISC-IP astrocytoma cells compared to RISC-IP astrocytes and miRNAs with a negative fold change are decreased in RISC-IP astrocytoma cells compared to RISC-IP astrocytes. (B) Hierarchical cluster heatmap showing all significantly expressed miRNAs (p<0.01) in four independent samples/cell type. Each row shows the relative expression level for a single miRNA and each column shows the expression level for a single sample. The red or green color indicates relative high or low expression, respectively. (C) Disease/disorders and (D) molecular and cellular functions associated with the miRNAs differentially expressed in RISC of U-87 astrocytoma cells compared to primary astrocytes with a Fisher's exact test p-value threshold set at 0.01 (yellow threshold line) using IPA software.

Comparison of miRNA levels in RISC of U-87 astrocytoma and primary astrocytes by microarray also showed that RISC miRNA levels were cell type specific (Figure 5). As previously described, all significantly (p<0.01) expressed miRNAs with a log2 fold change >±2 were denoted as miRNAs with key differences. Using cutoff, U-87 astrocytoma RISC contain a higher number of increased miRNA species compared to astrocyte RISC (Figure 5A). As such, miR-195, 10b, 29b, 19b, 34a and 455-3p were all increased in U-87 astrocytoma RISC as compared to primary astrocyte RISC (Figure 5A). MiR-181b was decreased with a log2 fold change >±2 in U-87 astrocytoma RISC as compared to primary astrocyte RISC (Figure 5A). Notably, 6 RISC-IP miRNAs had a significant log2 fold change >±3 (Figure 5A, Table 1). Only 7 miRNAs had a fold change >±2, yet many U-87 astrocytoma RISC-IP miRNA levels were still considered to be significantly different from levels in primary astrocyte RISC-IP miRNA (Figure 5B). When significant (p<0.01) miRNA levels for 4 independent U-87 astrocytoma and primary astrocyte RISC-IP samples were plotted on a hierarchical clustered heatmap, 50.8% of miRNAs (65 of 128) were increased in U-87 astrocytoma RISC compared to astrocyte RISC (Figure 5B). The remaining 49.2% of miRNAs (63 of 128) were decreased in U-87 astrocytoma RISC in comparison to astrocyte RISC (Figure 5B).

**Table 1:** Significantly expressed (p<0.01) RISC-immunoprecipitated miRNAs in human U-87 astrocytoma cells compared to primary human astrocytes with log2>3.

ID	Symbol	Fold Change	p-value
hsa-miR-195	MIR195	5.468	7.00E-07
hsa-miR-10b	MIR10B	5.273	2.91E-05
hsa-miR-29b	MIR29B	4.634	9.67E-07
hsa-miR-19b	MIR19B	3.320	6.17E-05
hsa-miR-34a	MIR34A	3.040	8.43E-09
hsa-miR-181b	MIR181B	−3.900	2.28E-06

These observations suggest U-87 astrocytoma cell RISC contain specific miRNAs that could potentially target tumor suppressor mRNA or act as oncogenes themselves resulting in increased proliferation, decreased cell death and tumor formation. Indeed, miR-19b located within the *mir-17–19b-1* cluster has been shown to function as an oncogene by targeting apoptotic factors that are activated in response to MYC overexpression, thereby allowing MYC to induce controlled cell proliferation [26]. In human glioblastoma samples, miR-10b was increased whereas brain-enriched miR-181b was decreased in human glioblastoma samples and in 10 human glioblastoma cell lines one of which included U-87 astrocytoma cells [53]. In support of increased miR-10b expression in gliomablastomas, another study showed that miR-10b was highly associated with higher grade gliomas suggesting that miR-10b may play a role in tumor invasiveness [54]. Further, miR-10b was also highly expressed in metastatic breast cancer cells and was shown to be involved in cell migration, invasion and metastasis [55].

In contrast to our observation of miR-195 levels being increased in U-87 astrocytoma RISC, miR-195 has been shown to be downregulated in various cancer cells including hepatocellular carcinoma [36], [37], [56]. MiR-195 was shown to play a fundamental role in cell cycle regulation and tumorigenesis where overexpression repressed phosphorylation of rentioblastoma (Rb)-E2F signalling downregulated mRNA targets such as cyclin D1, CDK6 and E2F3, thus suggesting miR-195 as a potential target for cancer therapy [57]. MiR-195 expression has not been specifically reported in brain cancers to date. Our results show miR-195 is in U-87 astrocytoma cell RISC as contrasted to primary astrocyte RISC, suggesting that U-87 astrocytoma RISC may be enriched in tumor suppressive miR-195 in an attempt to regulate the cell cycle and cell proliferation.

In our study, miR-34a was upregulated in U-87 astrocytoma RISC, however in other reports miR-34a was shown to be downregulated in cancer cells and tumors including colon cancer, leukemia, hepatocellular carcinoma and non-small cell lung cancer [58]–[60]. MiR-34a has been extensively studied in cancer and many studies point to miR-34a as a transcriptional target of p53 tumor suppressor whereby upregulation or activation of miR-34a contributes to p53-mediated apoptosis and G_1_ cell cycle arrest [61]–[63]. Other studies have shown that miR-34 overexpression also takes place independent of p53 upregulation [64], [65]. In recent years, miR-34a has been found to affect tumor cell apoptosis, senescence, proliferation and invasion [59], [63], [66]–[68]. In addition, a few mRNA targets of miR-34a are oncogenes such as MYC, CDK6, SIRT1 and c-Met [68]–[71]. In glioblastoma tumors, miR-34a was generally downregulated but when overexpressed, functioned to inhibit glioblastoma cell proliferation, survival, migration and invasion by targeting c-Met and Notch [72], [73]. MiR-34a is generally thought to be a tumor suppressor, however in our study and others, it has been found to be upregulated in several cancers including renal cell carcinoma [74], colon cancer [67] and hepatocellular carcinoma [75].

The 7 key IPA miRNAs with a log2 fold change >±2 in U-87 astrocytoma RISC compared to primary astrocyte RISC showed these miRNAs are associated with many diseases/disorders and have a role in specific molecular and cellular functions (Figure 5C, 5D). Specifically, U-87 astrocytoma RISC contain miRNAs that play a predominant role in inflammatory disease, cancer and genetic disorders and to a lesser extent in neurological disease and connective tissue disorders (Figure 5C, threshold set at p<0.01). In addition, U-87 astrocytoma RISC contain miRNAs that play a role in cellular compromise, development and growth and proliferation (Figure 5, threshold set at p<0.01 using Fisher's exact test). Not only are astrocytes important for neuronal survival and function, they contribute to the formation and preservation of a functional blood-brain barrier, control ionic and osmotic homeostasis [76], and play a role in the development and plasticity of the central nervous system by modifying the growth of axons and dendrites and regulating synapse formation [77]. They also play a central role in the control of the immune response and surveillance in the central nervous system [78], [79]. Indeed, our finding that U-87 astrocytoma RISC-specific miRNAs function to regulate inflammatory and immune responses to a greater extent than neurological processes suggests astrocytoma cells are characterized by RISC-associated dysregulated immune surveillance and inflammatory responses. Of interest, dicer-regulated miRNAs-222 and -339 have been reported to promote resistance to cytotoxic T-lymphocytes by down-regulating ICAM-1 [43].

A comparison of global miRNA expression between U-87 astrocytoma cells and primary astrocytes was not the primary focus of this study, yet this data has been included for completeness (Figure S2). In brief, there were 40 global miRNAs that had a log2 fold change greater than or equal to 2 in U-87 astrocytoma cells when compared to primary astrocytes (Figure S2A) with 8 of these global miRNAs having a significant log2 fold change >±3 (Figure S2A, Table 2). As with the cell type specific RISC-IP miRNAs, there was a relatively equal representation of increased and decreased miRNAs in the global cellular milieu of both cell types (Figure S2B). IPA of the 40 key global miRNAs with a log2 fold change >±2 in U-87 astrocytoma cells compared to primary astrocytes showed these miRNAs were predicted to be associated with the same, albeit a higher number of diseases/disorders than those miRNAs that were specifically within RISC (Figure S2C). Global miRNAs play a role in cellular compromise, cellular development and cellular growth and proliferation as do RISC miRNAs, however to a lesser extent than RISC-specific miRNAs (Figure S2D).

**Table 2:** Significantly expressed (p<0.01) global miRNAs in human U-87 astrocytoma cells compared to primary human astrocytes with log2>3.

ID	Symbol	Fold Change	p-value
hsa-miR-29b	MIR29B	6.214	3.52E-05
hsa-miR-29c	MIR29C	6.171	7.99E-05
hsa-miR-195	MIR195	4.491	5.75E-08
hsa-miR-20b	MIR20B	3.061	1.19E-07
hsa-miR-1280	MIR1280	−3.122	1.24E-06
hsa-miR-720	MIR720	−3.149	8.45E-08
hsa-miR-1826	MIR1826	−3.206	2.11E-09
hsa-miR-31	MIR31	−7.869	2.47E-03

### RISC contain mostly decreased levels of mRNAs in primary astrocytes and U-87 astrocytoma cells

**Figure 6:**
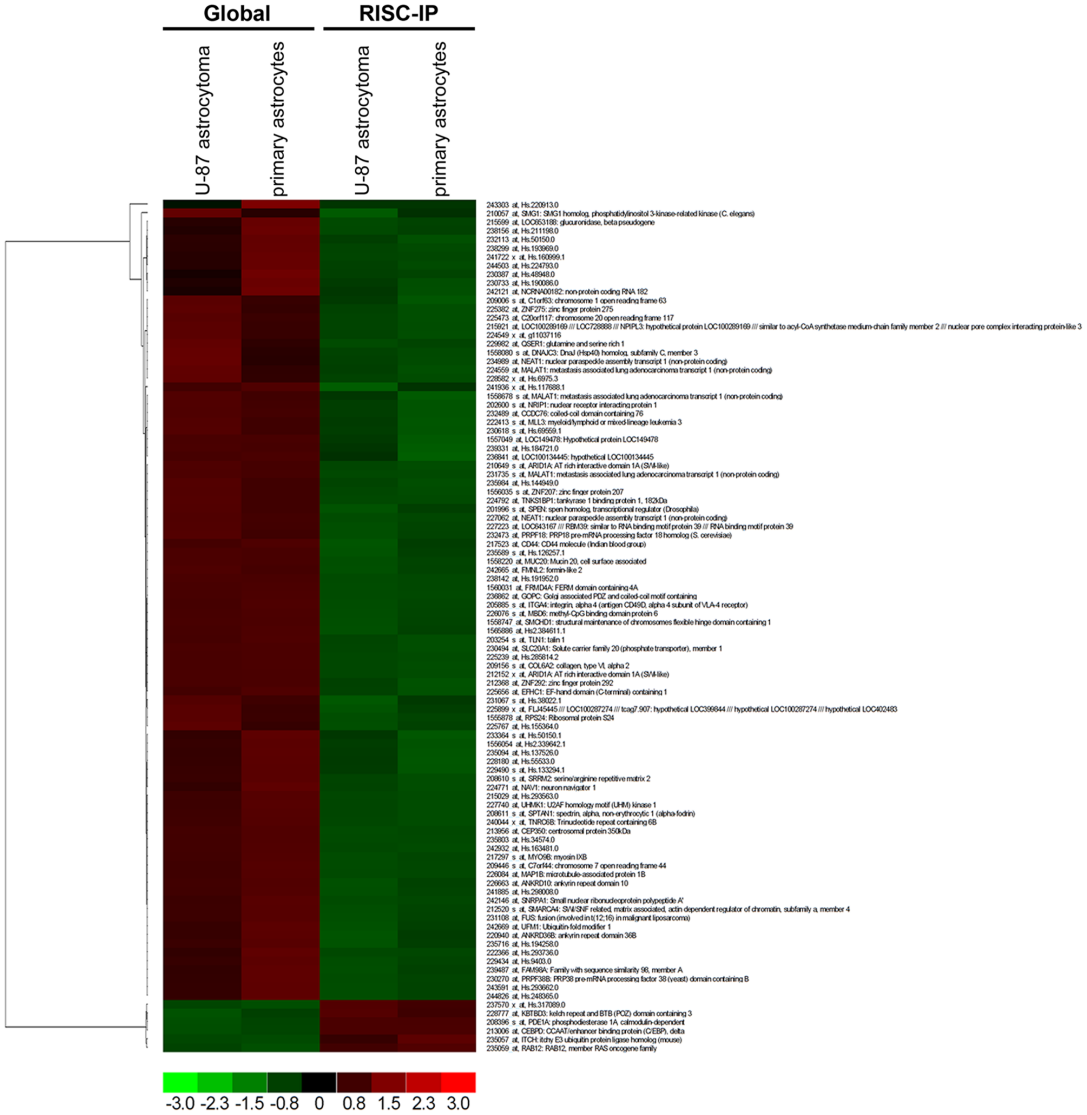
Global mRNA levels to RISC-IP mRNA levels in U-87 astrocytoma and primary astrocytes. A hierarchical heatmap comparing global mRNA levels to RISC-IP mRNA levels in U-87 astrocytoma and primary astrocytes. MRNAs included in the heatmap had a fold change >1.4 and were significantly expressed (p<0.01).

When global and RISC-IP gene expression in U-87 astrocytoma and primary astrocytes were analyzed on Affymetrix Human Genome U133A 2.0 Arrays, a slight variation in the specific global mRNA levels or RISC-IP mRNA levels between U-87 astrocytoma cells and primary astrocytes was observed. However, when significant (p<0.01) cell-specific expression values with a fold change >±1.4 (using the RMA algorithm) were grouped into a hierarchical clustered heat map of global versus RISC-IP sample sets, a unique profile of RISC-specific increased and decreased genes was observed (Figure 6). Relative to global gene expression, U-87 astrocytoma and primary astrocyte RISC contained 93.9% of mRNA that were significantly (p<0.01) decreased (92 of 98 total) and 6.1% of mRNA that were significantly (p<0.01) increased (6 of 98 total) (Figure 6), suggesting that the majority of mRNAs targeted to RISC are downregulated. Of the 98 genes expressed, 64 could be mapped to known genes in the Entrez gene database that encode enzymes, transcriptional regulators, transporters and other proteins (Table 3).

**Table 3:** RISC-immunoprecipitated mRNA compared to global cellular mRNA in U-87 astrocytoma cells and primary astrocytes with a fold change > ±1.8 (p<0.01).

Affymetrix ID	Symbol	Entrez Gene Name	Fold Change	Location	Type(s)
235059_at	RAB12	RAB12, member RAS oncogene family	1.850	Cytoplasm	enzyme
226663_at	ANKRD10	ankyrin repeat domain 10	−1.820	Nucleus	transcription regulator
227223_at	RBM39	RNA binding motif protein 39	−1.820	Nucleus	transcription regulator
231735_s_at	MALAT1	metastasis associated lung adenocarcinoma transcript 1 (non-protein coding)	−1.830	unknown	other
210057_at	SMG1	SMG1 homolog, phosphatidylinositol 3-kinase-related kinase (C. elegans)	−1.830	Cytoplasm	kinase
239487_at	FAM98A	family with sequence similarity 98, member A	−1.840	unknown	other
242146_at	SNRPA1	small nuclear ribonucleoprotein polypeptide A'	−1.860	Nucleus	other
227062_at	NEAT1 (includes EG:283131)	nuclear paraspeckle assembly transcript 1 (non-protein coding)	−1.890	unknown	other
1560031_at	FRMD4A	FERM domain containing 4A	−1.900	unknown	other
242669_at	UFM1	ubiquitin-fold modifier 1	−1.900	Cytoplasm	other
558080_s_at	DNAJC3	DnaJ (Hsp40) homolog, subfamily C, member 3	−1.910	Cytoplasm	other
242121_at	NCRNA00182	non-protein coding RNA 182	−1.930	unknown	other
210649_s_at	ARID1A	AT rich interactive domain 1A (SWI-like)	−1.980	Nucleus	transcription regulator
230270_at	PRPF38B	PRP38 pre-mRNA processing factor 38 (yeast) domain containing B	−2.050	unknown	other
234989_at	NEAT1 (includes EG:283131)	nuclear paraspeckle assembly transcript 1 (non-protein coding)	−2.100	unknown	other
230494_at	SLC20A1	solute carrier family 20 (phosphate transporter), member 1	−2.290	Plasma Membrane	transporter

**Figure 7:**
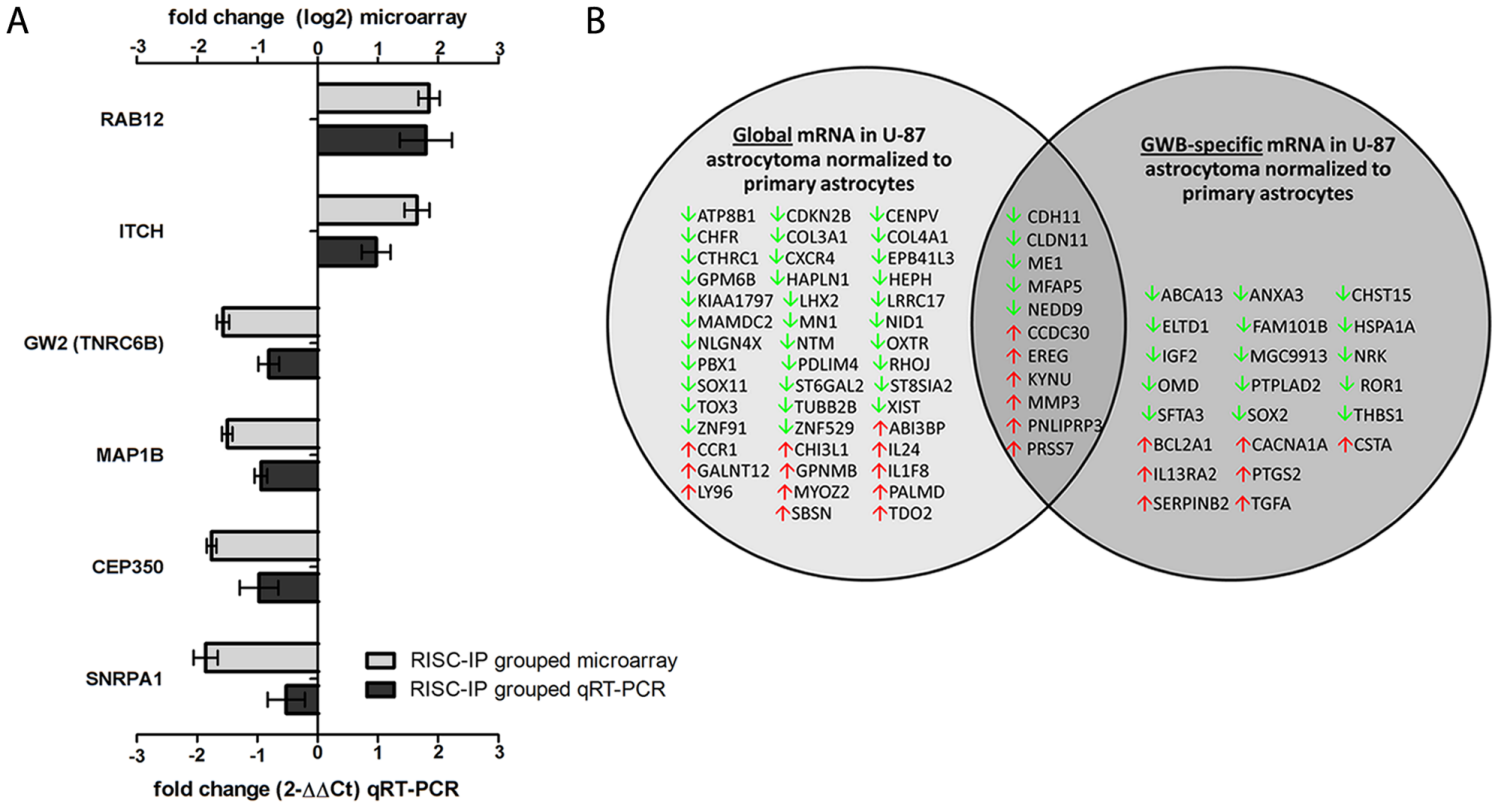
(**A**) MRNA microarray validation with qRT-PCR analysis in grouped RISC-IP U-87 astrocytoma and primary astrocytes samples. Grouped RISC-IP data were compared to the grouped global mRNA from U-87 astrocytoma and primary astrocytes samples. Eight mRNAs were selected from the grouped mRNA microarray dataset and examined by qRT-PCR. Fold change from the mRNA microarray are given by log2 values (left y-axis, light grey bars). Fold change from the qRT-PCR was determined using the 2^-ΔΔCt^ method and all mRNA expression values were normalized to the beta-actin endogenous control (right y-axis, dark grey bars). Error bars represent the standard deviation of the mean (SD). Importantly, the fold change (y-axis) cannot be directly compared between assays due to differences in calculation methods, but the general trend of up-regulation and down-regulation can be compared. (**B**) MRNAs in RISC compared to the global cellular milieu in U-87 astrocytoma cells. MRNA expression in U-87 astrocytoma cells were normalized to primary astrocytes mRNA expression. All mRNAs had a fold change >2.5 and were significantly expressed (p<0.01). Green and red arrows indicate decreased and increased levels respectively.

The gene expression microarray results for RISC-IP (U-87 astrocytoma and primary astrocyte) were validated by qRT-PCR with gene expression normalized to the beta-actin housekeeping gene (Figure 7A). As above, fold change values cannot be directly compared between microarray and qRT-PCR assays due to differences in calculation methods (log2 for microarray and 2^-ΔΔCt^ for qRT-PCR), however comparisons could be made based on the general trend (Figure 7A). The 6 mRNA cDNA examined by qRT-PCR included RAB12, ITCH, GW2 (TNRC6B), MAP1B, CEP350 and SNRPA1 (Figure 7A). In general, the qRT-PCR results for each of these mRNA cDNA correlated with the microarray results, whereby RAB12 and ITCH expression were both increased in RISC and GW2, MAP1B, CEP350 and SNRPA1 expression were decreased when compared to the global cellular mRNA expression levels (Figure 7A).

There were many decreased mRNAs that were shared between U-87 astrocytoma cells and primary astrocytes in RISC, as well as differences in RISC-specific mRNA levels (Figure S3). Differences in significant (p<0.01) mRNA levels between U-87 astrocytoma and astrocytes were mapped onto a hierarchical clustered heatmap with mRNAs that exhibited a fold change >±2.3 (Figure S3, Table 4). U-87 astrocytoma cell RISC contained 41 decreased mRNAs and 30 increased mRNAs out of a total of 74 mRNAs as compared to primary astrocytes (Figure S3) and include transmembrane receptors, growth factors, peptidases, transcription regulators, cytokines and other proteins as listed in Table 4. Globally, there were also specific mRNA level differences between U-87 astrocytoma cells and primary astrocytes (Figure S4, Table S2). In brief, 71 mRNAs were decreased and 30 mRNAs were increased in the global cellular fraction of U-87 astrocytoma cells as compared to primary astrocytes (Figure S4, p<0.01, log2 fold change >±2.3) and include enzymes, peptidases, cytokines, growth factors, G-protein coupled receptors, transcription regulators and others (Table S2).

**Table 4:** RISC-immunoprecipitated mRNA in human U-87 astrocytoma cells compared to RISC-immunoprecipitated mRNA in primary human astrocytes with a fold change >±2.6 (p<0.01).

Affymetrix ID	Symbol	Entrez Gene Name	Fold Change	Location	Type(s)
243036_at	CCDC30	coiled-coil domain containing 30	3.320	unknown	other
206172_at	IL13RA2	interleukin 13 receptor, alpha 2	3.140	Plasma Membrane	transmembrane receptor
205767_at	EREG	epiregulin	3.100	Extracellular Space	growth factor
207638_at	PRSS7	protease, serine, 7 (enterokinase)	2.830	Extracellular Space	peptidase
205681_at	BCL2A1	BCL2-related protein A1	2.780	Cytoplasm	other
205828_at	MMP3	matrix metallopeptidase 3 (stromelysin 1, progelatinase)	2.770	Extracellular Space	peptidase
1558945_s_at	CACNA1A	calcium channel, voltage-dependent, P/Q type, alpha 1A subunit	2.660	Plasma Membrane	ion channel
204971_at	CSTA	cystatin A (stefin A)	2.660	Cytoplasm	other
205016_at	TGFA	transforming growth factor, alpha	2.660	Extracellular Space	growth factor
217388_s_at	KYNU	kynureninase (L-kynurenine hydrolase)	2.650	Cytoplasm	enzyme
1558846_at	PNLIPRP3	pancreatic lipase-related protein 3	2.600	unknown	other
219134_at	ELTD1	EGF, latrophilin and seven transmembrane domain containing 1	−2.620	Plasma Membrane	G-protein coupled receptor
1553605_a_at	ABCA13	ATP-binding cassette, sub-family A (ABC1), member 13	−2.630	unknown	transporter
244050_at	PTPLAD2	protein tyrosine phosphatase-like A domain containing 2	−2.650	unknown	other
244741_s_at	MGC9913	hypothetical protein MGC9913	−2.700	unknown	other
227971_at	NRK	Nik related kinase	−2.760	unknown	kinase
202409_at	IGF2	insulin-like growth factor 2 (somatomedin A)	−2.870	Extracellular Space	growth factor
207173_x_at	CDH11	cadherin 11, type 2, OB-cadherin (osteoblast)	−2.900	Plasma Membrane	other
232060_at	ROR1	receptor tyrosine kinase-like orphan receptor 1	−2.900	Plasma Membrane	kinase
228979_at	SFTA3	surfactant associated 3	−2.940	unknown	other
228335_at	CLDN11	claudin 11	−2.950	Plasma Membrane	other
205907_s_at	OMD	osteomodulin	−3.250	Extracellular Space	other
213764_s_at	MFAP5	microfibrillar associated protein 5	−3.330	Extracellular Space	other
201110_s_at	THBS1	thrombospondin 1	−3.710	Extracellular Space	other

Comparison of mRNA levels in U-87 astrocytoma cell RISC and the global cellular fraction as compared to primary astrocytes provided a unique RISC mRNA profile, a unique global mRNA profile and a list of mRNAs that are shared between RISC and the global cellular milieu (Figure 7B). Overall, a greater number of mRNA species were unique to the global cellular fraction and were not found within the RISC in U-87 astrocytoma (p<0.01, log2 fold change >±2.5). Of these global-specific, 32 mRNA were decreased compared to 12 mRNAs that were increased (Figure 7B). RISC contained less mRNAs than the global cellular fraction and specifically contained 15 decreased mRNAs and 7 increased mRNAs (Figure 7B). Common to both fractions were 5 decreased mRNAs and 6 increased mRNAs (Figure 7B).

**Figure 8:**
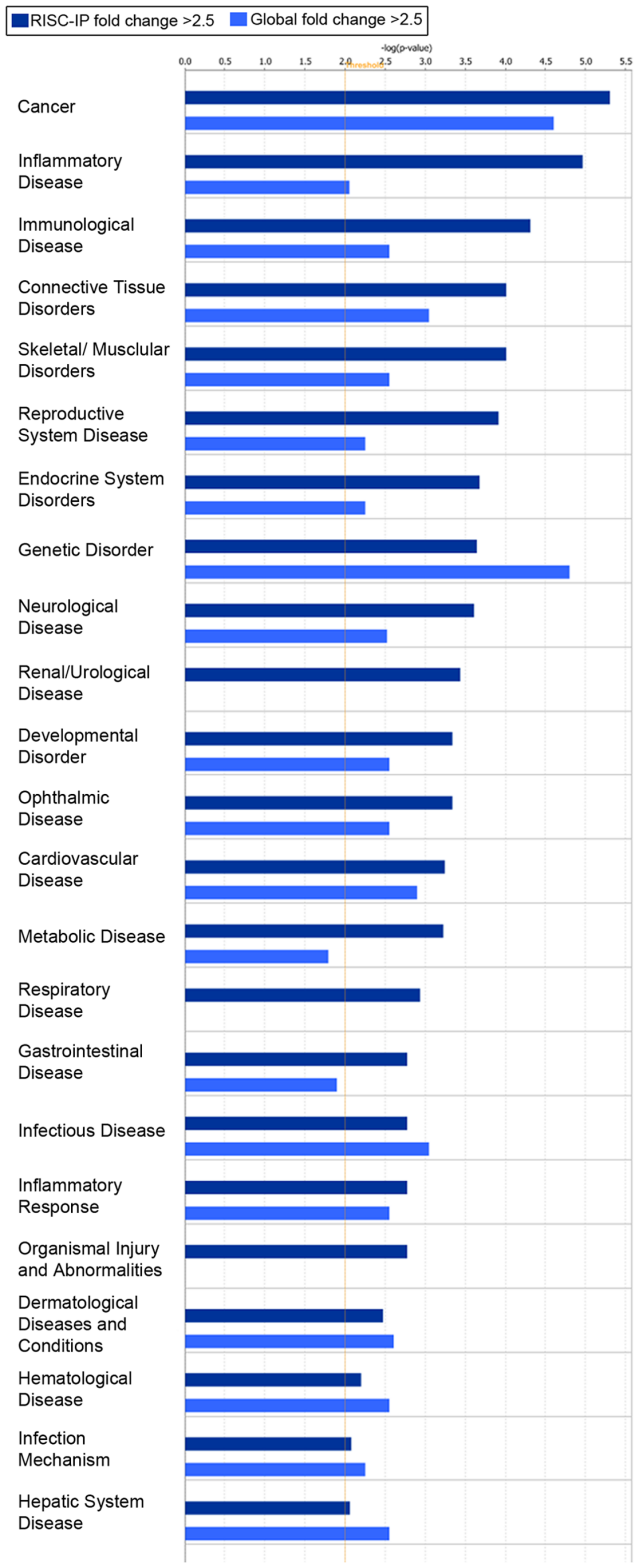
Disease and disorder representation of the mRNA in the RISC of U-87 astrocytoma cells compared to primary astrocytes and the mRNA in the global cellular milieu of U-87 astrocytoma cells compared to primary astrocytes. Bar charts display the relative number (-log(p-value)) of mRNAs with a fold change >2.5 and were considered significant (p<0.01). RISC-IP mRNA were indicated with dark blue bars and the global mRNA were indicated with light blue bars. The threshold (yellow lines) were set at p<0.01 and were calculated using Fischer's exact p-value test using IPA software.

**Figure 9:**
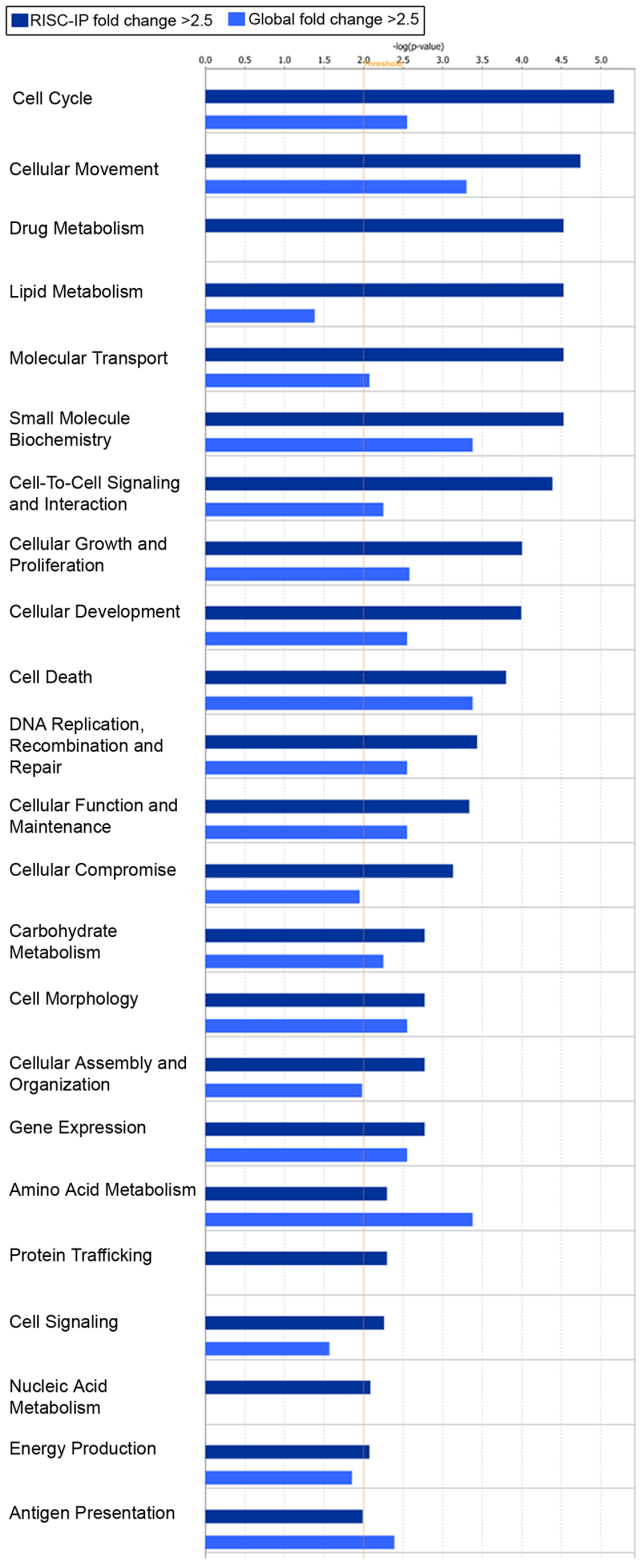
Molecular and cellular functional assessment of the mRNA in the RISC of U-87 astrocytoma cells compared to primary astrocytes and the mRNA in the global cellular milieu of U-87 astrocytoma cells compared to primary astrocytes. Bar charts display the relative number (-log(p-value)) of mRNAs with a fold change >2.5 and were considered significant (p<0.01). RISC-IP mRNA were indicated with dark blue bars and the global mRNA were indicated with light blue bars. The threshold (yellow lines) were set at p<0.01 and were calculated using Fischer's exact p-value test using IPA software.

Significant (p<0.01) mRNA levels with a log2 fold change >±2.5 in RISC and in the global cellular milieu of U-87 astrocytoma cells as compared to primary astrocytes were assessed using IPA for their predicted association with various diseases or disorders (Figure 8) and their predicted role in molecular and cellular functions (Figure 9). RISC were more highly enriched in mRNA involved in cancer, inflammatory, immunological and neurological disorders, among others (Figure 8). In comparison, the global cellular fraction was more highly enriched with mRNA that are involved in infectious diseases as well as hematological and dermatological disorders (Figure 8). U-87 astrocytoma RISC were more highly enriched in mRNA components that are involved in multiple cell functions and processes including the cell cycle, cellular growth and proliferation, DNA replication, DNA recombination and repair, and cellular assembly and organization, among others (Figure 9). In comparison, the global cellular fraction was more highly enriched with mRNA involved in antigen presentation and amino acid metabolism (Figure 9). Given that mRNAs important in cell cycle control and cancer are specifically enriched in the RISC within astrocytoma cells suggests the possibility that the RISC may be a future target for brain cancer therapies.

### Biological pathway analysis of miRNA and mRNA profile in U-87 astrocytoma RISC

**Figure 10:**
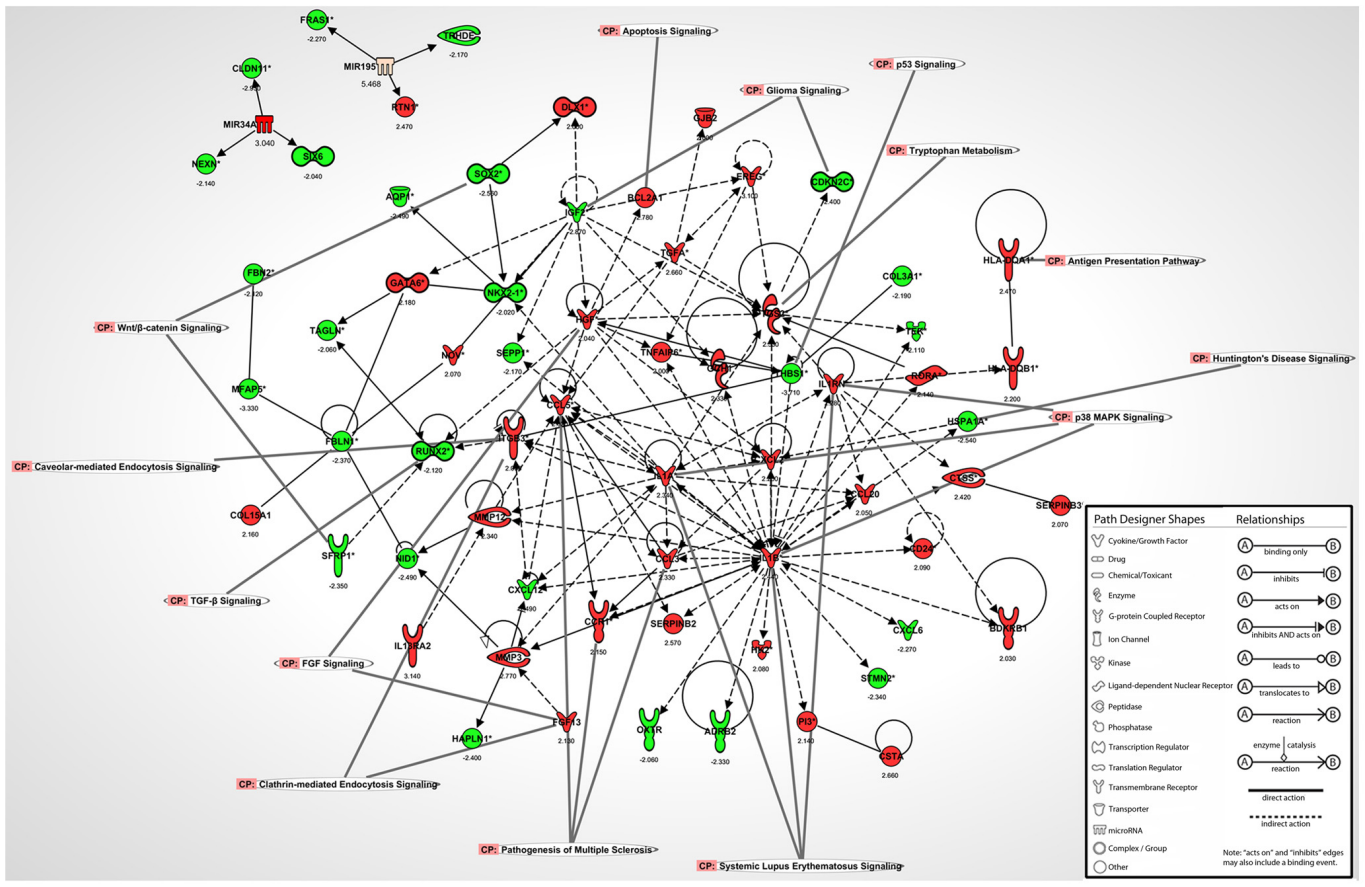
Biological pathway analysis showing the links between miRNA and mRNAs IP from RISC of U-87 astrocytoma cells compared to primary astrocytes using IPA software. All miRNA and mRNA were significantly expressed (p<0.01). MiRNA had a log2 fold change >3 and mRNA had a log2 fold change >2. Some key canonical pathways are indicated by labels.

RISC miRNA and mRNA microarray data was examined using IPA software to help appreciate the key mRNA-miRNA connections and biological pathways that were associated directly with the RISC and GW/P bodies (Figure 10). In this analysis, all selected miRNA had a fold change >±3 and all mRNA had a fold change >±2 (Figure 10). Each mRNA or miRNA was colored based upon the experimental expression level (i.e. green signified decreased expression and red specified increased expression) and the exact log2 fold change values were indicated below the molecule (Figure 10).

As noted above, miR-34a levels were increased in U-87 astrocytoma RISC compared to primary astrocyte RISC with a log2 fold change of 3.040 (Figure 3D, Table 1, p-value 8.43×10^−9^). In U-87 astrocytoma RISC, increased miR-34a levels corresponded specifically with the significantly (p<0.01) decreased levels of miR-34a predicted targets: claudin 11 (CLDN11*, −2.950), nexilin F actin binding protein (NEXN*, −2.140) and six homeobox 6 (SIX6, −2.040) which have roles in cell adhesion, permeability, migration and proliferation (Figure 10, Table 5). MiR-195 expression was also increased in U-87 astrocytoma GW/P bodies compared to primary astrocyte GW/P bodies with a log2 fold change of 5.468 (Figure 3D, Table 1, p-value 7.00×10^−7^). Increased miR-195 expression in U-87 astrocytoma RISC correlated with the significantly (p<0.01) decreased levels of miR-195 predicted targets: Fraser syndrome 1 (FRAS1*, −2.270) and thyrotropin-releasing hormone degrading enzyme (TRHDE, −2.170), which are involved in in cell communication and signalling (Figure 10, Table 6). MiR-195 is also predicted to silence reticulon 1 (RTN1*) which has a role in apoptosis, however the mRNA microarray results of our study show that RTN1* levels were increased by a log2 fold change of 2.470 in U-87 astrocytoma RISC, suggesting that miR-195 upregulation may have activated RTN1* (Figure 10, Table 6). Perhaps as in other cells, miR-195 function may have switched from repression to activation of target translation depending on the cell cycle status of astrocytoma cells [31], [80], [81]. Since U-87 astrocytoma cells rapidly proliferate in culture, it is unlikely that these cells had arrested and moved into quiescence (G_o_) thereby questioning the potential activation role of miR-195. Indeed, rather than being activated, RTN1* may be stabilized by another binding protein that would prevent miR-195 from binding to RTN1* to exert a repressive effect. Recent genome-wide studies have suggested there are two populations of miRNA, one that positively correlates with its target mRNAs and others that negatively correlate with cognate targets [82]–[84]. Thus, it is possible that miR-195 may have a dual function depending on the status of the mRNA target or where the mRNA is localized within the cell. This ‘ying-yang’ area of miRNA biology remains to be fully elucidated. Alternatively, RTN1* may not be a *bona fide* target of miR-195.

**Table 5:** Specific messenger RNA fold change linked to the increased levels of miR-34a in U-87 astrocytoma RISC.

Name	Entrez gene name	Fold Change	Role in cell	Canonical pathway/Biological process	Entrez gene summary
CLDN11	claudin 11	−2.950	adhesion, permeability, sealing in, formation, migration, proliferation, sealing	leukocyte extravasation signaling, tight junction signaling, cell adhesion, spermatogenesis, axon ensheathment, calcium-independent cell-cell adhesion	The protein encoded by this gene belongs to the claudin family of tight junction associated proteins and is a major component of central nervous system myelin that is necessary for normal CNS function. There is growing evidence that the protein determines the permeability between layers of myelin sheaths via focal adhesion and, with its expression highly regulated during development, may play an important role in cellular proliferation and migration. In addition, the protein is a candidate autoantigen in the development of autoimmune demyelinating disease.
NEXN	nexilin (F actin binding protein)	−2.140	migration	regulation of cell migration, regulation of cytoskeleton organization	NEXN is a filamentous actin (F-actin)-binding protein that localizes to focal contacts and may be involved in cell adhesion and migration (Ohtsuka et al., 1998 [PubMed 9832551]; Wang et al., 2005 [PubMed 15823560]).
SIX6	SIX homeobox 6	−2.040	proliferation	regulation of transcription, DNA-dependent, multicellular organism development, visual perception, organ morphogenesis	The protein encoded by this gene is a homeobox protein that is similar to the Drosophila ‘sine oculis’ gene product. This gene is found in a cluster of related genes on chromosome 14 and is thought to be involved in eye development. Defects in this gene are a cause of isolated microphthalmia with cataract type 2 (MCOPCT2).

**Table 6:** Specific messenger RNA fold change linked to increased levels of miR-195 in U-87 astrocytoma RISC.

Name	Entrez gene name	Fold Change	Role in cell	Canonical pathway/Biological process	Entrez gene summary
FRAS1	Fraser syndrome 1	−2.270	unknown	cell communication	This gene encodes an extracellular matrix protein that appears to function in the regulation of epidermal-basement membrane adhesion and organogenesis during development. Mutations in this gene cause Fraser syndrome, a multisystem malformation that can include craniofacial, urogenital and respiratory system abnormalities. Alternative splicing results in multiple transcript variants.
TRHDE	thyrotropin-releasing hormone degrading enzyme	−2.170	signaling	glutathione metabolism, proteolysis, signal transduction, cell-cell signaling	This gene encodes a member of the peptidase M1 family. The encoded protein is an extracellular peptidase that specifically cleaves and inactivates the neuropeptide thyrotropin-releasing hormone.
RTN1	reticulon 1	2.470	apoptosis	signal transduction, neuron differentiation	This gene belongs to the family of reticulon encoding genes. Reticulons are associated with the endoplasmic reticulum, and are involved in neuroendocrine secretion or in membrane trafficking in neuroendocrine cells. Alternatively spliced transcript variants encoding different isoforms have been identified. Multiple promoters rather than alternative splicing of internal exons seem to be involved in this diversity.

Beyond the 6 mRNAs targeted by miR-34a and miR-195, there were many mRNAs that were increased and decreased in U-87 astrocytoma RISC that were directly (solid black arrows) or indirectly (dashed black arrows) connected through another molecule within the same pathway (Figure 10). Central mRNAs with decreased expression in which 4 or more other mRNAs converged included insulin-like growth factor 2 (IGF2, −2.870), fibulin 1 (FBLN1*, −2.370), thrombospondin 1 (THBS1*, −3.710) and chemokine C-X-C motif ligand 12 (CXCL12*, −2.490) all of which have functions in apoptosis, proliferation and migration (Figure 10). Central mRNAs with increased expression in which 4 or more other mRNAs converged included epiregulin (EREG, 3.100), chemokine C-C motif ligand 3 (CCL3, 2.330), chemokine C-X-C motif ligand 2 (CXCL2, 2.250), interleukin 1 alpha (IL1A*, 2.340), interleukin 1 beta (IL1B*, 2.440), interleukin 1 receptor antagonist (IL1RN*, 2.280), tumor necrosis factor alpha induced protein factor 6 (TNFAIP6, 2.000), transforming growth factor alpha (TGFA*, 2.660), prostaglandin-endoperoxide synthase 2 (PTGS2*, 2.530), matrix metallopeptidase 3 (MMP3, 2.770) and matrix metallopeptidase 12 (MMP12, 2.340) which have functions in apoptosis, migration, proliferation and invasion (Figure 10). The mRNAs that are involved in key canonical pathways are indicated by labels and include Systemic Lupus Erythematosus signalling, p38 MAPK signalling, clathrin-mediated endocytosis signalling, pathogenesis of multiple sclerosis and others (Figure 10). The majority (>90%) of these mRNAs localized to U-87 astrocytoma RISC are associated with tumorigenesis and cancer (label not shown on biological pathway to avoid figure crowding), which suggests the miRNA-loaded RISC is a key regulator of mRNAs involved in cancer and cell cycle regulation in U-87 astrocytoma cells.

The hypothesis was the RISC-specific miRNA and mRNA components will not only differ between malignant and non-malignant cells of the same cell lineage, but will also differ between cells originating from different organs. In this study, evidence is provided that RISC contains miRNA and mRNA species whose levels are increased or decreased compared to the global cellular miRNA and mRNA levels and differ in miRNA and mRNA composition between cell types, although the overall functional impact of these differences remains to be elucidated. In order to go beyond reporting log2 values of various miRNA and mRNA species within GW/P bodies/RISC compared to the whole cell fraction for the two human cell types used, our study employed the use of IPA software to contextualize the dataset in terms of disease and molecular pathways. Limitations of IPA analysis of microarray data are acknowledged and other interpretations and conclusions with respect to our data are possible. Nevertheless, IPA remains a useful method to assign disease/pathway relevance to multiple miRNA and mRNA species across cell lines and from multiple experiments without endeavouring to perform time- and cost-intensive functional analysis on an individual miRNA or mRNA. The IPA predicted targets and specific functions of mRNA and miRNAs within the RISC must be validated by *in vitro* or *in vivo* functional studies.

In this study, the GW/P body/RISC protein complex was immunoprecipitated using the well-documented and widely used GW/P body prototype human serum (18033) containing antibodies to GW182 and hAgo2. The human serum used for IP has been documented to contain GW182 antibodies but did not contain antibodies to the other GW family members, GW2 and GW3. In addition, being a polyclonal serum, 18033 also contains antibodies to hAgo2 but there is no evidence that it targets Ago1, Ago3 or Ago4. However, at least two reports have shown that antibodies to Ago2 are generally cross-reactive with other Argonaute proteins (1, 3 and 4) most likely because they have ∼90% sequence homology [85], [86]. This suggests that 18033 may also cross-immunoprecipitate Ago1, Ago3 and Ago4 proteins. Thus, in addition to Ago2, the data derived in our experiments likely encompassed extensive heterogeneity of Ago components believed to be embedded in the RISC.

The current study provides a methodological framework that produced a set of unique profiles from human primary astrocytes and U-87 astrocytoma cells in which to start building the RISC-miRNA and RISC-mRNA component repository. Further RISC and GW/P body RNA component profiling is required.

## Supporting Information

Figure S1 Global versus RISC-IP miRNA probe reporter signal intensity for (A) U-87 astrocytoma cells and (B) primary human astrocytes.
(2.88 MB TIF)

Figure S2 Global miRNA expression in human U-87 astrocytoma cells compared to primary human astrocytes.
(2.14 MB TIF)

Figure S3 Hierarchical cluster heatmap of significant mRNA expression in primary astrocytes and U-87 astrocytoma cells isolated from RISC-IP. MRNAs included in the heatmap had a fold change >2.3 (p<0.01).
(2.36 MB TIF)

Figure S4 Hierarchical cluster heatmap of significant mRNA expression in primary astrocytes and U-87 astrocytoma cells isolated global total RNA samples. MRNAs included in the heatmap had a fold change >2.3 (p<0.01).
(0.63 MB TIF)

Table S1 Forward and reverse primers used for validation of gene expression and miRNA expression by qRT-PCR analysis.
(0.04 MB DOC)

Table S2 Global mRNA levels in human U-87 astrocytoma cells compared to primary human astrocytes with a fold change greater than 2.3 with p<0.01.
(0.17 MB DOC)
