# Supplementary figures and images for: The MicroRNA and MessengerRNA Profile of the RNA-Induced Silencing Complex in Human Primary Astrocyte and Astrocytoma Cells
Source: PLoS One. 2010 Oct 18;5(10):e13445. doi: 10.1371/journal.pone.0013445 (PMC2956662; doi:10.1371/journal.pone.0013445)

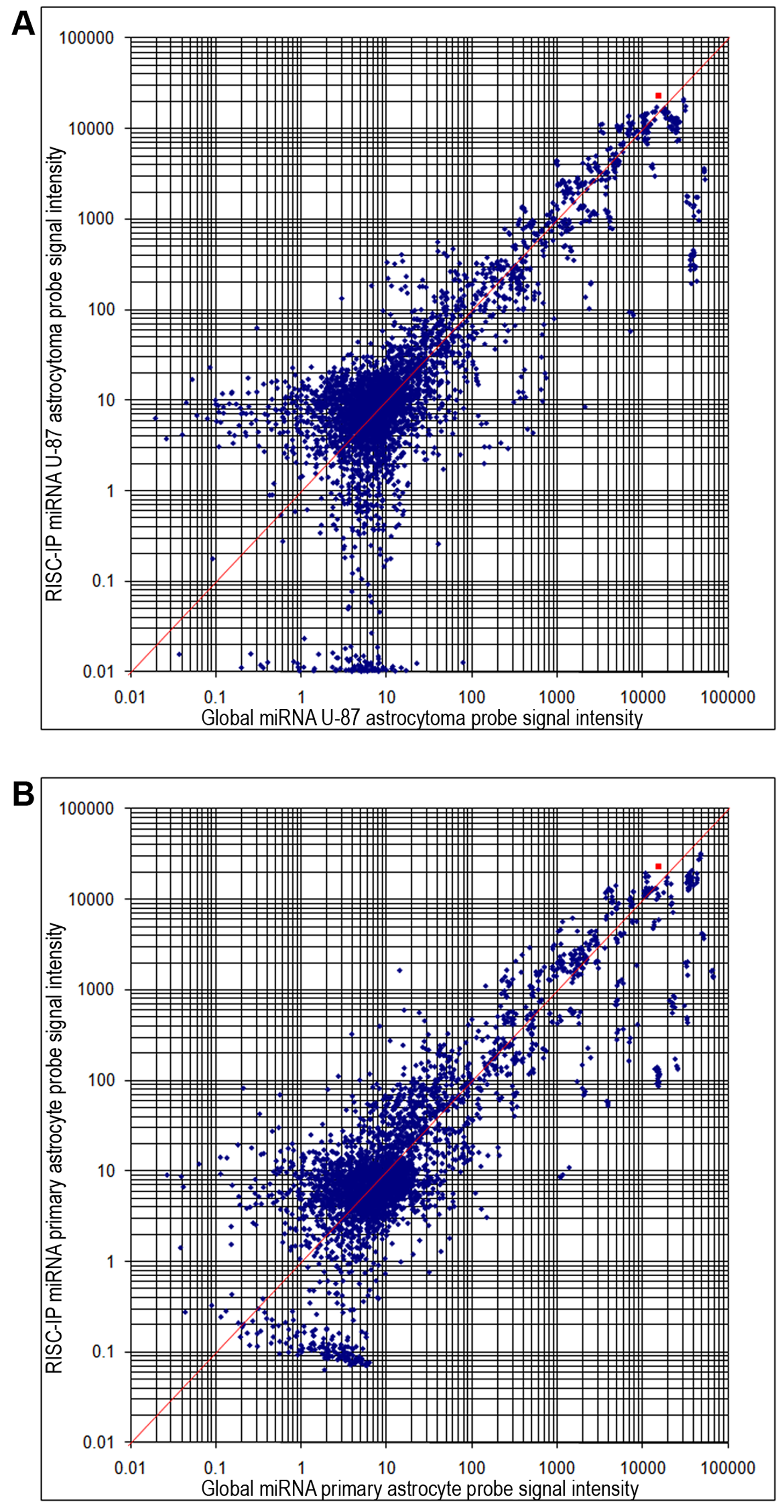

Supplement: Figure S1 — Global versus RISC-IP miRNA probe reporter signal intensity for (A) U-87 astrocytoma cells and (B) primary human astrocytes. (2.88 MB TIF) [file pone.0013445.s001.tif]

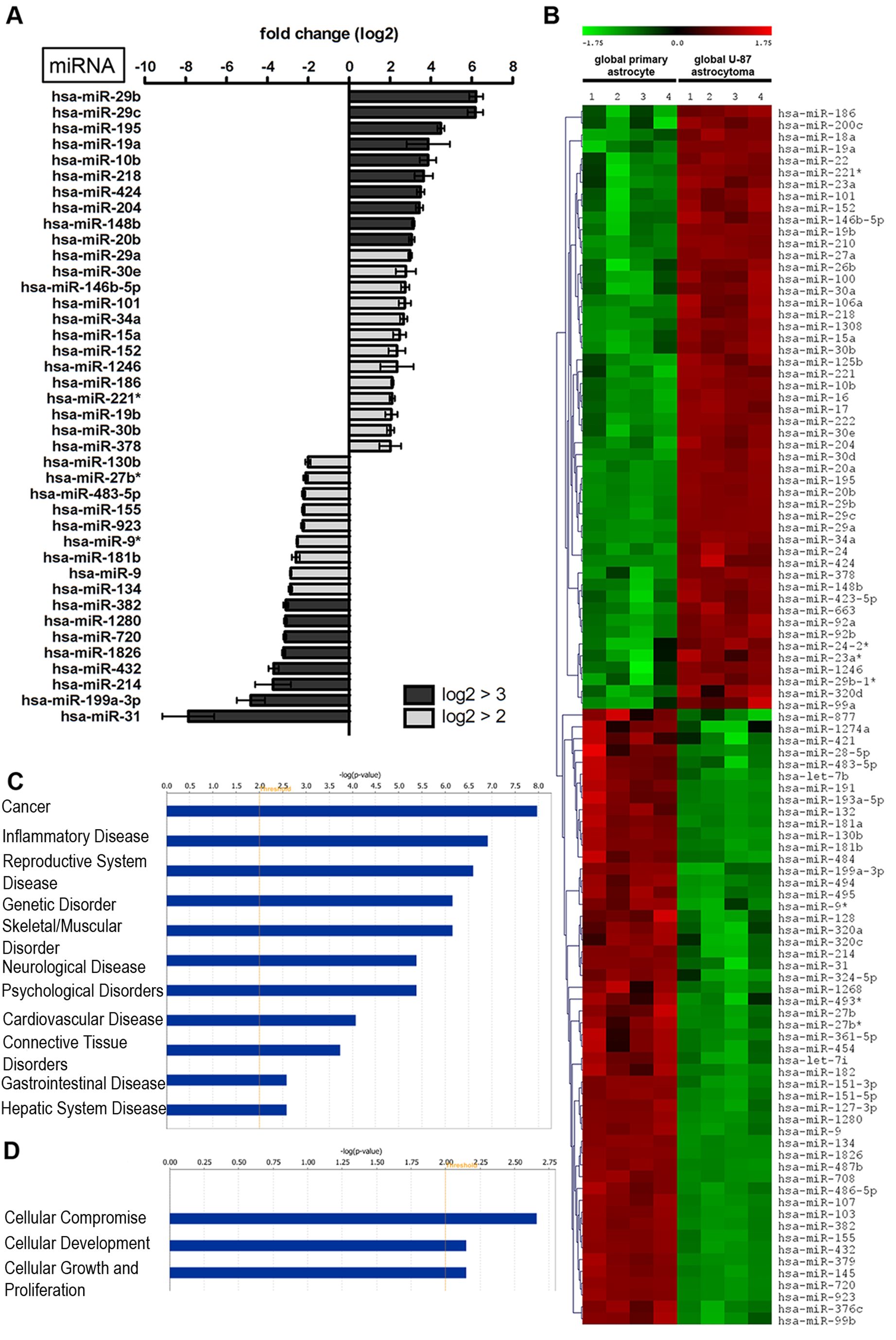

Supplement: Figure S2 — Global miRNA expression in human U-87 astrocytoma cells compared to primary human astrocytes. (2.14 MB TIF) [file pone.0013445.s002.tif]

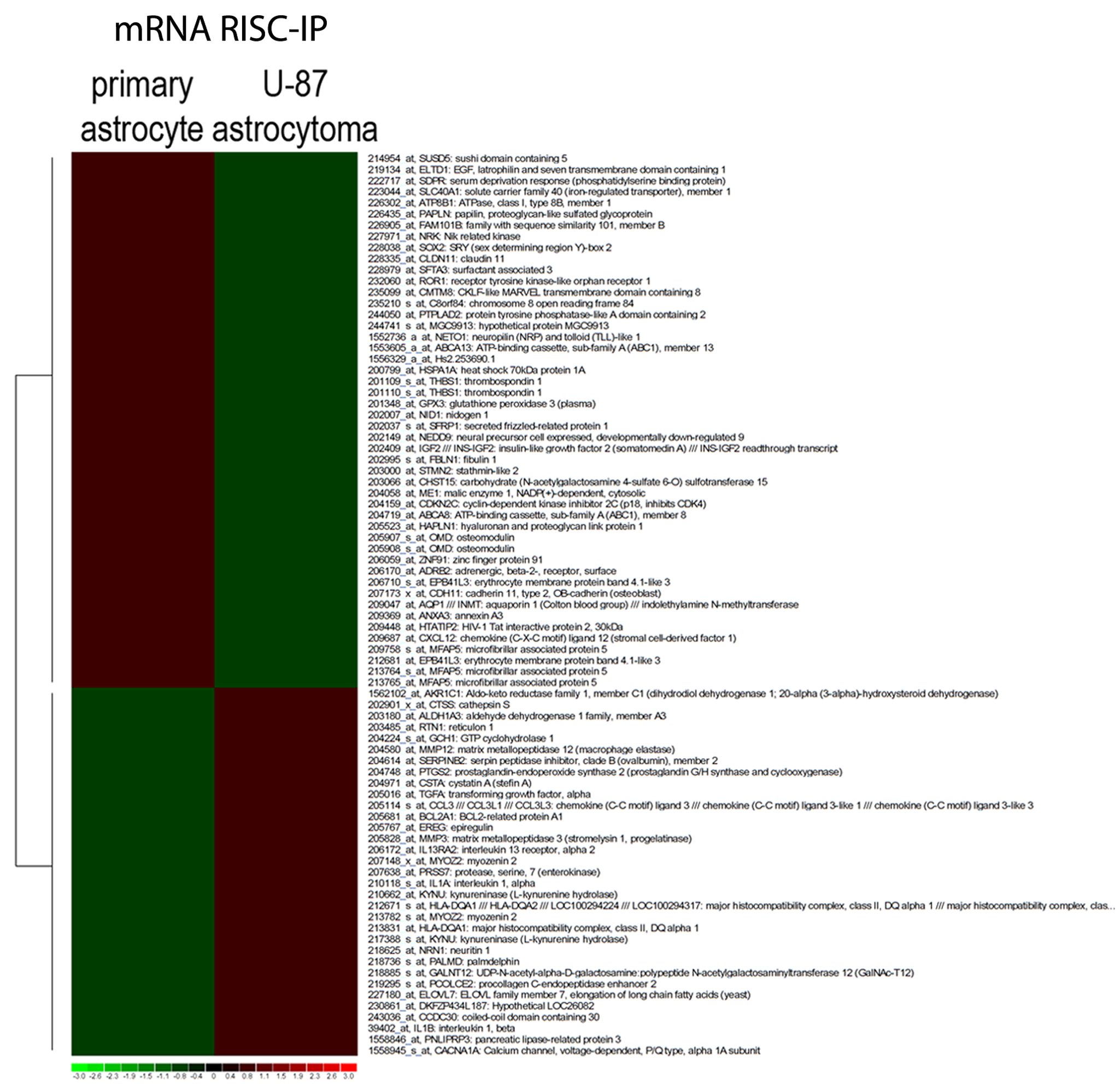

Supplement: Figure S3 — Hierarchical cluster heatmap of significant mRNA expression in primary astrocytes and U-87 astrocytoma cells isolated from RISC-IP. MRNAs included in the heatmap had a fold change >2.3 (p<0.01). (2.36 MB TIF) [file pone.0013445.s003.tif]

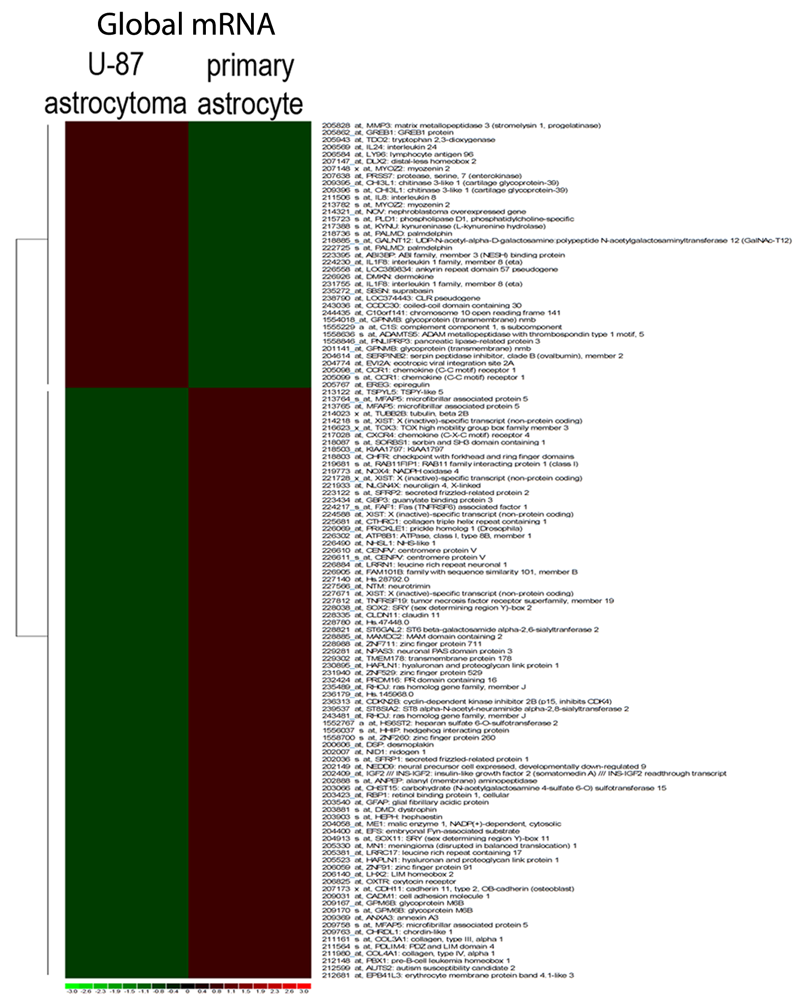

Supplement: Figure S4 — Hierarchical cluster heatmap of significant mRNA expression in primary astrocytes and U-87 astrocytoma cells isolated global total RNA samples. MRNAs included in the heatmap had a fold change >2.3 (p<0.01). (0.63 MB TIF) [file pone.0013445.s004.tif]
