# Supplementary material for: The MicroRNA and MessengerRNA Profile of the RNA-Induced Silencing Complex in Human Primary Astrocyte and Astrocytoma Cells
Source: PLoS One. 2010 Oct 18;5(10):e13445. doi: 10.1371/journal.pone.0013445 (PMC2956662; doi:10.1371/journal.pone.0013445)
Supplement: Table S1 — Forward and reverse primers used for validation of gene expression and miRNA expression by qRT-PCR analysis. (0.04 MB DOC) [file pone.0013445.s005.doc]

**Table S1: Forward and reverse primers used for validation of gene expression and miRNA expression by qRT-PCR analysis**.

| **Primer Name** | **Forward Probe Sequence (5’→3’)** | **Reverse Probe Sequence (5’→3’)** |
| --- | --- | --- |
| β-actin | CTGGAACGGTGAAGGTGACA | AAGGGACTTCCTGTAACAATGCA |
| RAB12 | gaagtgccaaggggatcata | tggcacttgcttcacagaac |
| ITCH | GTAGCCTCACCATGAAATCACA | CGATAACTGTAAGGGGTTGCTT |
| GW2 (TNRC6B) | AGGAAATTGGAGGAATGTGAGTG | GGATGTCTGACCTACTGTGCT |
| MAP1B | AAGCTGAGAGGTCCCTTATGT | CTTCGTCTTCGATTAGCTCCAG |
| CEP350 | AGAACGGAATATACGGAGCTGT | TTAGTCGTAACGCTTTTGGATCA |
| SNRPA1 | TGCTACGTTAGACCAGTTTGATG | CCCTCACCTATACGGCATATTCT |
| RNU6B | TGACACGCAAATTCGTGAAG | Universal qPCR primer |
| hsa-miR-29b | TAGCACCATTTGAAATCAGTGTT | Universal qPCR primer |
| hsa-miR-29c | TAGCACCATTTGAAATCGGTTA | Universal qPCR primer |
| hsa-miR-195 | GATAGCAGCACAGAAATATTGGC | Universal qPCR primer |
| hsa-miR-10b | TACCCTGTAGAACCGAATTTGTG | Universal qPCR primer |
| hsa-miR-20b | CAAAGTGCTCATAGTGCAGGTAG | Universal qPCR primer |
| hsa-miR-181b | TCATTGCTGTCGGTGGGT | Universal qPCR primer |
| hsa-miR-1280 | CACCGCTGCCACCCA | Universal qPCR primer |
| hsa-miR-1826 | TCGACACTTCGAACGCAATA | Universal qPCR primer |
| hsa-miR-432 | TGGAGTAGGTCATTGGGTGG | Universal qPCR primer |
| hsa-miR-19b | GCAAATCCATGCAAAACTGA | Universal qPCR primer |
| hsa-miR-455-3p | GCAGTCCATGGGCATATACAC | Universal qPCR primer |
| hsa-miR-27b | TTCACAGTGGCTAAGTTCTGC | Universal qPCR primer |
