# Supplementary material for: The MicroRNA and MessengerRNA Profile of the RNA-Induced Silencing Complex in Human Primary Astrocyte and Astrocytoma Cells
Source: PLoS One. 2010 Oct 18;5(10):e13445. doi: 10.1371/journal.pone.0013445 (PMC2956662; doi:10.1371/journal.pone.0013445)
Supplement: Table S2 — Global mRNA levels in human U-87 astrocytoma cells compared to primary human astrocytes with a fold change greater than 2.3 with p<0.01. (0.17 MB DOC) [file pone.0013445.s006.doc]

**Table S2: Global mRNA levels in human U-87 astrocytoma cells compared to primary human astrocytes with a fold change greater than 2.3 with p<0.01.**

| **Affymetrix ID** | **Symbol** | **Entrez Gene Name** | **Fold Change** | **Location** | **Type(s)** |
| --- | --- | --- | --- | --- | --- |
| 217388_s_at | KYNU | kynureninase (L-kynurenine hydrolase) | 3.340 | Cytoplasm | enzyme |
| 1554018_at | GPNMB | glycoprotein (transmembrane) nmb | 3.220 | Plasma Membrane | enzyme |
| 213782_s_at | MYOZ2 | myozenin 2 | 2.990 | Cytoplasm | other |
| 1558846_at | PNLIPRP3 | pancreatic lipase-related protein 3 | 2.990 | unknown | other |
| 205828_at | MMP3 | matrix metallopeptidase 3 (stromelysin 1, progelatinase) | 2.940 | Extracellular Space | peptidase |
| 209395_at | CHI3L1 | chitinase 3-like 1 (cartilage glycoprotein-39) | 2.930 | Extracellular Space | enzyme |
| 223395_at | ABI3BP | ABI family, member 3 (NESH) binding protein | 2.890 | unknown | other |
| 205098_at | CCR1 | chemokine (C-C motif) receptor 1 | 2.890 | Plasma Membrane | G-protein coupled receptor |
| 205767_at | EREG | epiregulin | 2.880 | Extracellular Space | growth factor |
| 243036_at | CCDC30 | coiled-coil domain containing 30 | 2.810 | unknown | other |
| 231755_at | IL1F8 | interleukin 1 family, member 8 (eta) | 2.740 | Extracellular Space | cytokine |
| 206569_at | IL24 | interleukin 24 | 2.700 | Extracellular Space | cytokine |
| 205943_at | TDO2 | tryptophan 2,3-dioxygenase | 2.700 | Cytoplasm | enzyme |
| 218736_s_at | PALMD | palmdelphin | 2.680 | unknown | other |
| 207638_at | PRSS7 | protease, serine, 7 (enterokinase) | 2.680 | Extracellular Space | peptidase |
| 235272_at | SBSN | suprabasin | 2.640 | unknown | other |
| 218885_s_at | GALNT12 | UDP-N-acetyl-alpha-D-galactosamine:polypeptide N-acetylgalactosaminyltransferase 12 (GalNAc-T12) | 2.510 | Cytoplasm | enzyme |
| 206584_at | LY96 | lymphocyte antigen 96 | 2.500 | Plasma Membrane | other |
| 1555229_a_at | C1S | complement component 1, s subcomponent | 2.490 | Extracellular Space | peptidase |
| 238790_at | LOC374443 | CLR pseudogene | 2.430 | unknown | other |
| 215723_s_at | PLD1 | phospholipase D1, phosphatidylcholine-specific | 2.410 | Cytoplasm | enzyme |
| 204614_at | SERPINB2 | serpin peptidase inhibitor, clade B (ovalbumin), member 2 | 2.410 | Extracellular Space | other |
| 214321_at | NOV | nephroblastoma overexpressed gene | 2.360 | Extracellular Space | growth factor |
| 1558636_s_at | ADAMTS5 | ADAM metallopeptidase with thrombospondin type 1 motif, 5 | 2.350 | Extracellular Space | peptidase |
| 244435_at | FAM196A | family with sequence similarity 196, member A | 2.350 | unknown | other |
| 204774_at | EVI2A | ecotropic viral integration site 2A | 2.340 | Plasma Membrane | transmembrane receptor |
| 207147_at | DLX2 | distal-less homeobox 2 | 2.330 | Nucleus | transcription regulator |
| 211506_s_at | IL8 | interleukin 8 | 2.330 | Extracellular Space | cytokine |
| 226926_at | DMKN | dermokine | 2.310 | unknown | other |
| 226558_at | LOC389834 | ankyrin repeat domain 57 pseudogene | 2.310 | unknown | other |
| 205862_at | GREB1 | growth regulation by estrogen in breast cancer 1 | 2.300 | Cytoplasm | other |
| 219489_s_at | NXN | nucleoredoxin | -2.300 | Nucleus | enzyme |
| 232424_at | PRDM16 | PR domain containing 16 | -2.300 | Nucleus | transcription regulator |
| 235518_at | SLC8A1 | solute carrier family 8 (sodium/calcium exchanger), member 1 | -2.300 | Plasma Membrane | transporter |
| 229302_at | TMEM178 | transmembrane protein 178 | -2.300 | unknown | other |
| 226490_at | NHSL1 | NHS-like 1 | -2.310 | unknown | other |
| 202036_s_at | SFRP1 | secreted frizzled-related protein 1 | -2.310 | Plasma Membrane | transmembrane receptor |
| 218087_s_at | SORBS1 | sorbin and SH3 domain containing 1 | -2.310 | Plasma Membrane | other |
| 224217_s_at | FAF1 | Fas (TNFRSF6) associated factor 1 | -2.320 | Nucleus | other |
| 213122_at | TSPYL5 | TSPY-like 5 | -2.320 | unknown | other |
| 1558700_s_at | ZNF260 | zinc finger protein 260 | -2.320 | Nucleus | other |
| 203881_s_at | DMD | dystrophin | -2.330 | Plasma Membrane | other |
| 202409_at | IGF2 | insulin-like growth factor 2 (somatomedin A) | -2.330 | Extracellular Space | growth factor |
| 209763_at | CHRDL1 | chordin-like 1 | -2.350 | Extracellular Space | other |
| 200606_at | DSP | desmoplakin | -2.350 | Plasma Membrane | other |
| 229281_at | NPAS3 | neuronal PAS domain protein 3 | -2.350 | Nucleus | other |
| 202888_s_at | ANPEP | alanyl (membrane) aminopeptidase | -2.360 | Plasma Membrane | peptidase |
| 1556037_s_at | HHIP | hedgehog interacting protein | -2.360 | Plasma Membrane | other |
| 226884_at | LRRN1 | leucine rich repeat neuronal 1 | -2.360 | unknown | other |
| 209369_at | ANXA3 | annexin A3 | -2.370 | Cytoplasm | enzyme |
| 209031_at | CADM1 | cell adhesion molecule 1 | -2.370 | Plasma Membrane | other |
| 228988_at | ZNF711 | zinc finger protein 711 | -2.390 | Nucleus | other |
| 212599_at | AUTS2 (includes EG:26053) | autism susceptibility candidate 2 | -2.400 | unknown | other |
| 204400_at | EFS | embryonal Fyn-associated substrate | -2.400 | Cytoplasm | other |
| 219773_at | NOX4 | NADPH oxidase 4 | -2.400 | Cytoplasm | enzyme |
| 226069_at | PRICKLE1 | prickle homolog 1 (Drosophila) | -2.410 | Nucleus | other |
| 219681_s_at | RAB11FIP1 | RAB11 family interacting protein 1 (class I) | -2.410 | Cytoplasm | other |
| 228038_at | SOX2 | SRY (sex determining region Y)-box 2 | -2.420 | Nucleus | transcription regulator |
| 227812_at | TNFRSF19 | tumor necrosis factor receptor superfamily, member 19 | -2.430 | Plasma Membrane | transmembrane receptor |
| 552767_a_at | HS6ST2 | heparan sulfate 6-O-sulfotransferase 2 | -2.440 | Extracellular Space | enzyme |
| 226905_at | FAM101B | family with sequence similarity 101, member B | -2.450 | unknown | other |
| 223122_s_at | SFRP2 | secreted frizzled-related protein 2 | -2.450 | Plasma Membrane | transmembrane receptor |
| 203540_at | GFAP | glial fibrillary acidic protein | -2.460 | Cytoplasm | other |
| 223434_at | GBP3 | guanylate binding protein 3 | -2.470 | Cytoplasm | enzyme |
| 203066_at | CHST15 | carbohydrate (N-acetylgalactosamine 4-sulfate 6-O) sulfotransferase 15 | -2.480 | Plasma Membrane | enzyme |
| 203423_at | RBP1 | retinol binding protein 1, cellular | -2.480 | Extracellular Space | transporter |
| 217028_at | CXCR4 | chemokine (C-X-C motif) receptor 4 | -2.500 | Plasma Membrane | G-protein coupled receptor |
| 218503_at | KIAA1797 | KIAA1797 | -2.500 | unknown | other |
| 231940_at | ZNF529 | zinc finger protein 529 | -2.510 | unknown | other |
| 204058_at | ME1 | malic enzyme 1, NADP(+)-dependent, cytosolic | -2.520 | Cytoplasm | enzyme |
| 216623_x_at | TOX3 | TOX high mobility group box family member 3 | -2.520 | unknown | other |
| 203903_s_at | HEPH | hephaestin | -2.540 | Plasma Membrane | transporter |
| 239537_at | ST8SIA2 | ST8 alpha-N-acetyl-neuraminide alpha-2,8-sialyltransferase 2 | -2.540 | Cytoplasm | enzyme |
| 206059_at | ZNF91 | zinc finger protein 91 | -2.540 | Nucleus | transcription regulator |
| 211564_s_at | PDLIM4 | PDZ and LIM domain 4 | -2.550 | Cytoplasm | other |
| 218803_at | CHFR | checkpoint with forkhead and ring finger domains | -2.590 | Nucleus | enzyme |
| 226302_at | ATP8B1 | ATPase, class I, type 8B, member 1 | -2.600 | Plasma Membrane | transporter |
| 206140_at | LHX2 | LIM homeobox 2 | -2.600 | Nucleus | transcription regulator |
| 228885_at | MAMDC2 | MAM domain containing 2 | -2.600 | Extracellular Space | other |
| 228821_at | ST6GAL2 | ST6 beta-galactosamide alpha-2,6-sialyltranferase 2 | -2.600 | Cytoplasm | enzyme |
| 202007_at | NID1 | nidogen 1 | -2.610 | Extracellular Space | other |
| 206825_at | OXTR | oxytocin receptor | -2.610 | Plasma Membrane | G-protein coupled receptor |
| 226611_s_at | CENPV | centromere protein V | -2.630 | Nucleus | other |
| 212681_at | EPB41L3 | erythrocyte membrane protein band 4.1-like 3 | -2.640 | Plasma Membrane | other |
| 205381_at | LRRC17 | leucine rich repeat containing 17 | -2.660 | unknown | other |
| 209170_s_at | GPM6B | glycoprotein M6B | -2.730 | Plasma Membrane | other |
| 205330_at | MN1 | meningioma (disrupted in balanced translocation) 1 | -2.750 | Nucleus | other |
| 235489_at | RHOJ | ras homolog gene family, member J | -2.750 | Cytoplasm | enzyme |
| 224588_at | XIST | X (inactive)-specific transcript (non-protein coding) | -2.760 | Nucleus | other |
| 228335_at | CLDN11 | claudin 11 | -2.770 | Plasma Membrane | other |
| 211161_s_at | COL3A1 | collagen, type III, alpha 1 | -2.780 | Extracellular Space | other |
| 202149_at | NEDD9 | neural precursor cell expressed, developmentally down-regulated 9 | -2.780 | Nucleus | other |
| 212148_at | PBX1 | pre-B-cell leukemia homeobox 1 | -2.780 | Nucleus | transcription regulator |
| 225681_at | CTHRC1 | collagen triple helix repeat containing 1 | -2.820 | Extracellular Space | other |
| 214023_x_at | TUBB2B | tubulin, beta 2B | -2.860 | Cytoplasm | other |
| 221933_at | NLGN4X | neuroligin 4, X-linked | -2.880 | Plasma Membrane | enzyme |
| 236313_at | CDKN2B | cyclin-dependent kinase inhibitor 2B (p15, inhibits CDK4) | -2.890 | Nucleus | transcription regulator |
| 227566_at | NTM | neurotrimin | -2.890 | Plasma Membrane | other |
| 213764_s_at | MFAP5 | microfibrillar associated protein 5 | -2.900 | Extracellular Space | other |
| 207173_x_at | CDH11 | cadherin 11, type 2, OB-cadherin (osteoblast) | -3.000 | Plasma Membrane | other |
| 204913_s_at | SOX11 | SRY (sex determining region Y)-box 11 | -3.060 | Nucleus | transcription regulator |
| 205523_at | HAPLN1 | hyaluronan and proteoglycan link protein 1 | -3.070 | Extracellular Space | other |
| 211980_at | COL4A1 | collagen, type IV, alpha 1 | -3.380 | Extracellular Space | other |
